# NK cells and monocytes modulate primary HTLV-1 infection

**DOI:** 10.1371/journal.ppat.1010416

**Published:** 2022-04-04

**Authors:** Ramona Moles, Sarkis Sarkis, Veronica Galli, Maria Omsland, Maria Artesi, Massimiliano Bissa, Katherine McKinnon, Sophia Brown, Vincent Hahaut, Robyn Washington-Parks, Joshua Welsh, David J. Venzon, Anna Gutowska, Melvin N. Doster, Matthew W. Breed, Kristin E. Killoran, Joshua Kramer, Jennifer Jones, Marcin Moniuszko, Anne Van den Broeke, Cynthia A. Pise-Masison, Genoveffa Franchini

**Affiliations:** 1 Animal Models and Retroviral Vaccines Section, Center for Cancer Research, National Cancer Institute, Bethesda, Maryland, United States of America; 2 Laboratory of Experimental Hematology, Institut Jules Bordet, Université Libre de Bruxelles, Brussels, Belgium; 3 Unit of Animal Genomics, GIGA, Université de Liège, Liège, Belgium; 4 Vaccine Branch Flow Cytometry Core, National Cancer Institute, National Institutes of Health, Bethesda, Maryland, United States of America; 5 Translational Nanobiology Section, Center for Cancer Research, National Cancer Institute, Bethesda, Maryland, United States of America; 6 Biostatistics and Data Management Section, Center for Cancer Research, National Cancer Institute, Bethesda, Maryland, United States of America; 7 Laboratory Animal Sciences Program, Leidos Biomedical Research Inc., Frederick National Laboratory, Frederick, Maryland, United States of America; 8 Department of Allergology and Internal Medicine, Medical University of Bialystok, Bialystok, Poland; Imperial College London, UNITED KINGDOM

## Abstract

We investigated the impact of monocytes, NK cells, and CD8^+^ T-cells in primary HTLV-1 infection by depleting cell subsets and exposing macaques to either HTLV-1 wild type (HTLV-1_WT_) or to the HTLV-1_p12KO_ mutant unable to infect replete animals due to a single point mutation in *orf-I* that inhibits its expression. The *orf-*I encoded p8/p12 proteins counteract cytotoxic NK and CD8^+^ T-cells and favor viral DNA persistence in monocytes. Double NK and CD8^+^ T-cells or CD8 depletion alone accelerated seroconversion in all animals exposed to HTLV-1_WT_. In contrast, HTLV-1_p12KO_ infectivity was fully restored only when NK cells were also depleted, demonstrating a critical role of NK cells in primary infection. Monocyte/macrophage depletion resulted in accelerated seroconversion in all animals exposed to HTLV-1_WT_, but antibody titers to the virus were low and not sustained. Seroconversion did not occur in most animals exposed to HTLV-1_p12KO._ *In vitro* experiments in human primary monocytes or THP-1 cells comparing HTLV-1_WT_ and HTLV-1_p12KO_ demonstrated that *orf-I* expression is associated with inhibition of inflammasome activation in primary cells, with increased CD47 “don’t-eat-me” signal surface expression in virus infected cells and decreased monocyte engulfment of infected cells. Collectively, our data demonstrate a critical role for innate NK cells in primary infection and suggest a dual role of monocytes in primary infection. On one hand, *orf-I* expression increases the chances of viral transmission by sparing infected cells from efferocytosis, and on the other may protect the engulfed infected cells by modulating inflammasome activation. These data also suggest that, once infection is established, the stoichiometry of *orf-I* expression may contribute to the chronic inflammation observed in HTLV-1 infection by modulating monocyte efferocytosis.

## Author summary

The immune cells that inhibit or favor HTLV-1 infection are still unknown and their identification is critical for understanding viral pathogenesis and for the development of an effective HTLV-1 vaccine. Neutralizing antibodies are produced in natural HTLV-1 infection, but their impact is likely hampered by the virus’s ability to be transmitted from cell to cell via the virological synapse, cellular conduits, and biofilms. By depleting specific immune cell subsets in blood, we found that NK cells play a critical role in the containment of early HTLV-1 infection. Moreover, transient depletion of monocytes/macrophages results in early, but not sustained seroconversion, suggesting that early engagement of monocytes may be necessary for long-term productive infection. The engulfment of apoptotic T-cells infected by HTLV-1 may represent a viral strategy to persist in the host since the viral proteins encoded by *orf-I* and *orf-II* affect the function of receptors and proteins involved in efferocytosis. These results suggest that effective HTLV-1 vaccines must also elicit durable innate responses able to promptly clear virus invasion of monocytes through engulfment of infected T-cells to avoid the establishment of a vicious cycle that leads to chronic inflammation.

## Introduction

The Human T-cell Leukemia Virus type-1 (HTLV-1) retrovirus causes Adult-T-cell Leukemia/Lymphoma and the neurodegenerative disorder HTLV-1 Associated Myelopathy/Tropical Spastic Paraparesis [1–6]. HTLV-1 integrates in the human genome [3,7,8] and establishes a durable DNA viral reservoir in T-cells and myeloid cells [9–19]. While viral integration is well-documented in T-cells, it remains uncertain if this process occurs in monocytes due to the ability of monocytes/macrophages to engulf HTLV-1 infected T-cells. HTLV-1 transmission occurs via cell to cell contact through virological synapses, cellular conduits, and biofilms [20–27]; no cell-free virus is detected in the blood of infected individuals. Immune-dysregulation is a common feature in HTLV-1 infection [16,28–34], and life-long virus persistence occurs even in the face of strong innate and adaptive responses [35–40], likely linked to the ability of the virus to counteract host responses.

The non-structural protein product p12, expressed from the HTLV-1 *orf-I* by alternative splicing, is post-translationally cleaved to the p8 protein [41–43]. Both p8 and p12 are dispensable in viral replication *in vitro* [44–47], however they are essential for viral infectivity/persistence *in vivo* [38,44]. The p12 and p8 proteins counteract natural killer (NK) cells [48,49] and CD8^+^ cytotoxic T-cell (CTL) [38] responses *in vitro* and augment T-cell proliferation [50,51] and viral transmission [24,52,53]. The p12 protein localizes in the endoplasmic reticulum (ER) and Golgi apparatus via a retention signal that, once removed, allows the cleaved p8 protein to localize to the cellular surface [54–56]. In the ER, p12 interacts with the heavy chain of major histocompatibility complex class I (MHC-I) and reroutes it to proteasomal degradation [56], causing a decrease in cell surface MHC-I expression and suboptimal recognition of infected cells by cytotoxic T-cells [38,48]. Moreover, p12 also downregulates ICAM-1 and ICAM-2, reducing NK cell recognition [49]. The p12 protein interacts with calreticulin and calnexin to promote calcium release, activate the nuclear factor of T-cells (NFAT) [57], and stimulate T-cell proliferation [58,59]. Furthermore, its binding to both of the interleukin 2 receptor (IL2-R) β and γ chains in the ER promotes STAT5 activation and reduces the IL-2 requirement for T-cell growth [51].

The p8 protein is recruited to the immunological synapse and, upon antigen stimulation, downregulates proximal T-cell receptor (TCR) signaling, thus causing T-cell anergy [52]. The p8 protein is transferred from cell to cell by cellular conduits and increases T-cell adhesiveness and HTLV-1 transmission [24,26,38,60,61]. Recently, p8 has also been shown to bind to the vasodilator-stimulator phosphoprotein (VASP) that promotes actin filament elongation [62], an important step in phagocytosis [63]. Both p12 and p8 are necessary for efficient HTLV-1 infectivity in macaques, as demonstrated by a prior study in which an HTLV-1 molecular clone was engineered by site-specific mutagenesis to ablate expression of both proteins encoded by *orf-I* (HTLV-1_p12KO_) and was demonstrated to be unable to infect macaques [44]. In humans, genetic mutations surrounding the p12/p8 cleavage sites altering the p12/p8 ratio are associated with lower viral burden [38], a documented predictor of disease development [64–68].

In the current work, our investigation into the relative importance of CD8, NK cells, and myeloid cells in the early phases of viral transmission revealed an unexpected, critical role for NK cells in restricting viral infection and that the decrease in monocytes/macrophages early in infection on one hand facilitates seroconversion, yet on the other renders seroconversion unsustainable. *In vitro* studies demonstrated that *orf-I* expression is also necessary for dampening inflammasome activation in monocytes and for upregulating the CD47 “don’t-eat-me” signal on infected cells. These seemingly contrasting results suggest the hypothesis that *orf-I* may favor viral persistence by sparing a portion of HTLV-1 infected cells from efferocytosis, and, possibly, by hijacking the clearance of the engulfed infected cells via inhibition of inflammasome activation.

## Results

### Critical role of Natural Killer cells in HTLV-1 transmission

Prior data demonstrated that both *orf-I* products p8 and p12 are required to protect infected T-cells from CTL-mediated killing *in vitro* and viral persistence *in vivo* [38], suggesting the possibility that the CTL response could be key to inhibiting viral infection. However, *orf-I* expression is also required to escape NK recognition and killing *in vitro* and for viral DNA persistence in monocytes [44,49]. To dissect which of these immune responses is critical to inhibit viral transmission and/or early viral replication *in vivo*, we administered monoclonal antibodies that deplete either CD8^+^β lymphocytes (CD8β255R1) or both CD8^+^ lymphocytes and NK cells (M-T807R1). CD8β255R1 recognizes the CD8 α/β chains that selectively deplete CD8 T-cells without also depleting non-CD8 T-cell populations that express CD8, such as natural killer cells [69]. In comparison, M-T807R1 recognizes the α/α chains of CD8^+^ lymphocytes and NK cells [70,71]. To deplete monocytes and tissue macrophages, we exposed animals to clodronate delivered in liposomes [72] prior to exposure to virus-producing irradiated cells infected with either HTLV-1_WT_ or HTLV-1_p12KO_ (**Table 1**). Controls for these experimental treatments included macaques exposed to irradiated uninfected cells following treatment with M-T807R1 (two animals), macaquses exposed to irradiated HTLV-1_WT_ cells following treatment with an isotype IgG control (two animals), or macaques exposed to irradiated HTLV-1_WT_ cells following treatment with liposomes (three animals; **S1A Fig**). M-T807R1 effectively depleted CD8^+^ cells (**S1B–S1E Fig**), total NKG2A^+^, and, in part, the NKG2A^+^CD16^+^ subsets (**S1C and S1D** and **S2A Figs**) as expected. The IgG isotype and liposome treatments had no effect on CD8^+^ cells (**S1E, S2B and S2C Figs**) or monocytes (**S1F Fig**) respectively, and neither treatment affected other cell populations (**S2B and S2C Fig**). Likewise, neither treatment with IgG nor liposome exacerbated the frequency of HTLV-1_WT_ infection in macaques, as approximately half of the animals exposed to HTLV-1 fully seroconverted as shown previously [44], and were positive by nested PCR for viral DNA in blood (**S1G Fig,** bottom panel). Only animal ZJ11 had low antibodies titers (1:100) to p24Gag in ELISA at week 12, and the two animals that scored positive by Western blot at week 21 (H28P and HFM) had undetectable ELISA titers (**S1H Fig**). Prior treatment and history for the animals enrolled in this study are summarized in **S1 Table.**

**Table 1:** Study design and animals.

Group	Treatment	Virus Inoculation	Number of Animals (n)	Animal ID
α-CD8/NK Ctrl uninfected	M-T807R1	none	2	H28W
				ZN19
IgG WT	IgG1 OKT3	HTLV-1_WT_	2	H28P
				HFM
α-CD8 WT	CD8β255R1	HTLV-1_WT_	3	ZL54
				ZN25
				ZN28
α-CD8 p12KO	CD8β255R1	HTLV-1_p12KO_	4	ZL49
				ZN17
				ZN12
				H861A
α-CD8/NK WT	M-T807R1	HTLV-1_WT_	3	ZM32
				ZN36
				ZN01
α-CD8/NK p12KO	M-T807R1	HTLV-1_p12KO_	4	HXR
				HRC
				ZN23
				H16X
Liposome WT	Encapsome	HTLV-1_WT_	3	ZJ11
				ZJ58
				H28J
Clodronate WT	Clodrosome	HTLV-1_WT_	5	ZJ22
				Zi51
				HPM
				P651
				R257
Clodronate p12KO	Clodrosome	HTLV-1_p12KO_	5	M616
				P205
				P206
				P212
				P213

**Fig 1:**
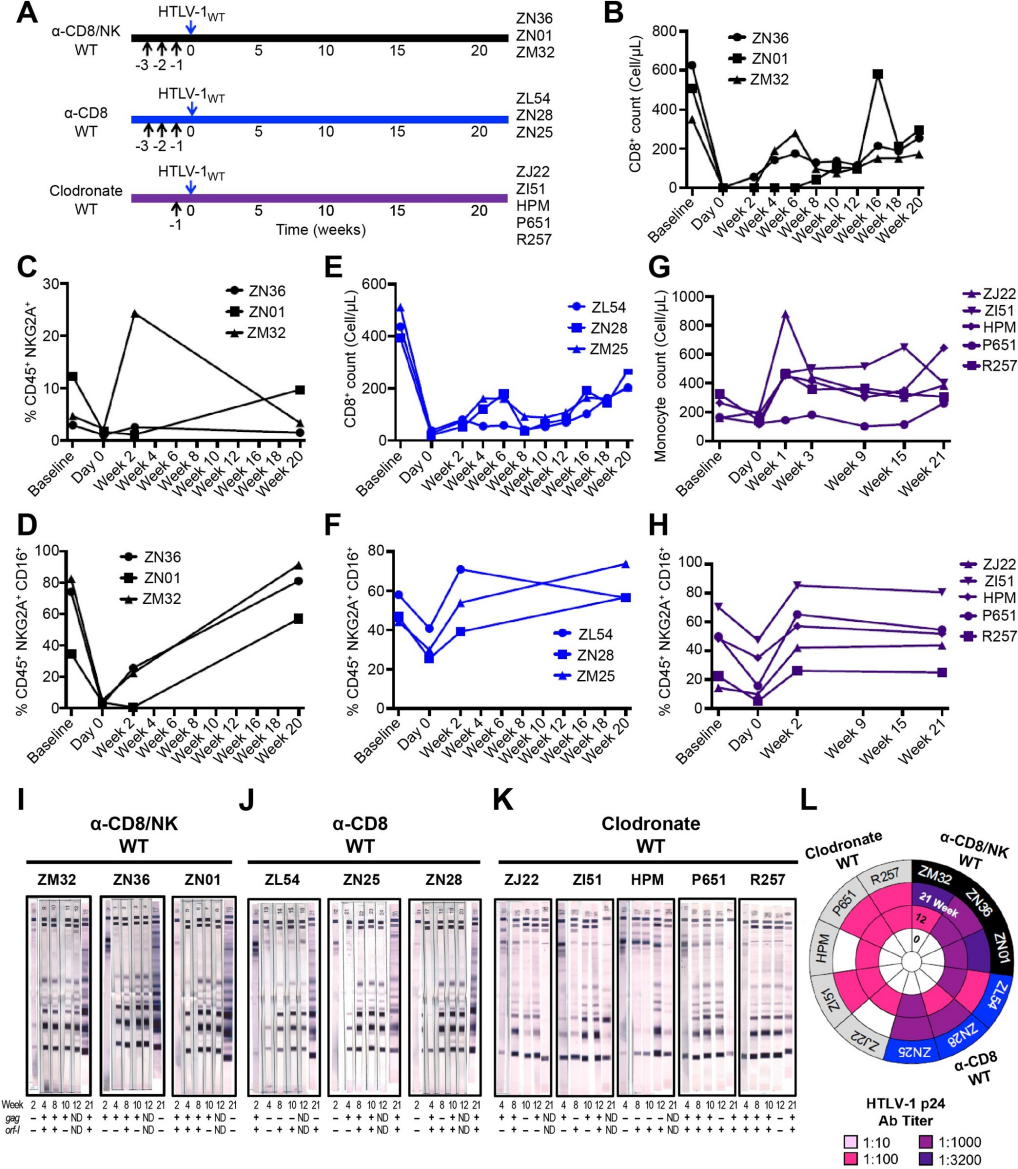
Exacerbation of HTLV-1_WT_ infectivity by depletion of immune cell subsets. (**A**) Schematic of study design. Black arrows represent the day of treatments (M-T807R1, CD8ß255R1, or Clodrosome). Blue arrows indicate inoculation day of the lethally irradiated 729.6 cells lymphoblastoid B-cell lines producing HTLV-1_WT_. (**B,E**) Absolute CD8^+^ T-cell numbers shown before (baseline), during (day 0), and after (weeks 2, 4, 6, 8, 10, 12, 16, 18, and 20) the administration of M-T807R1 and CD8β255R1. A complete blood count (CBC) of CD8^+^ was performed in the (**B**) α-CD8/NK WT and (**E**) α-CD8 WT groups inoculated with HTLV-1_WT_. (**C**) The frequency of NKG2A^+^ cells identified as Singlets/Live/CD45^+^/CD3^-^CD20^-^/NKG2A^+^ was measured before (baseline), during (day 0), and after (weeks 2 and 20) CD8^+^ cell depletion in peripheral blood of the α-CD8/NK WT group. (**D,F,H**) Frequency of NKG2A^+^CD16^+^ cells identified as Singlets/Live/CD45^+^/CD3^-^CD20^-^/NKG2A^+^CD16^+^ before (baseline), during (day 0), and after (weeks 2 and 20) inoculation for the (**D**) α-CD8/NK WT, (**F**) α-CD8 WT, and (**H**) Clodronate WT groups. (**G**) Absolute monocyte cell numbers shown before (baseline), during (day 0), and after (weeks 1, 3, 9, 15, and 21) the administration of Clodrosome and inoculation with HTLV-1_WT_. (**I,J,K**) Sera from the inoculated macaques belonging to (**I**) α-CD8/NK WT, (**J**) α-CD8 WT, and (**K**) Clodronate WT groups were assayed at weeks 2, 4, 8, 10, 12, and 21 for reactivity to HTLV-1 antigens using the kit from HTLV Blot 2.4 Western Blot Assay (MP Diagnostics, Singapore). The animal ID, inoculated viruses, and treatment are indicated above each sample. The week of sera collection is indicated below each western blot strip. Moreover, below each sample a nested PCR amplifying the *gag* (top row) and *orf-I* (bottom row) genes was performed in the blood of each animal throughout the course of the study. Positive amplification of either *gag* or *orf-I* is symbolized by (+); absence of amplification is symbolized by (-). (**L**) HTLV-1 p24Gag antibody titer was measured for macaques belonging to α-CD8/NK WT, α-CD8 WT, and Clodronate WT groups at weeks 0, 12, and 21. Dilutions of 1:10, 1:100, 1:1000, and 1:3200 were used and color-coded as reported in the figure.

Macaques exposed to HTLV-1_WT_ (**Fig 1A**) following treatment with M-T807R1 showed depletion of CD8^+^ T-cells, NKG2A^+^, and NKG2A^+^CD16^+^ [73] (**Figs 1B–1D** and **S3A**).

Macaques exposed to HTLV-1_WT_ following treatment with CD8β255R1 also showed depletion of CD8^+^ T-cells (**Figs 1E** and **S3B**), and furthermore had partial depletion of NKG2A^+^, and particularly the CD16^+^ subset (**Figs 1F** and **S3B**). This result is not unexpected since CD8^+^ makes up a portion of NK cells in Indian rhesus macaques [73]. Clodronate treatment decreased total monocyte counts (**Figs 1G** and **S3C**) and, surprisingly, also partially decreased the NKG2A^+^CD16^+^ subset (**Figs 1H** and **S3C**). All treatments enhanced HTLV-1_WT_ infection in 100% of the animals, compared to the 50% observed in replete animals. Because the viral load in this model system is <0.02%, we measured viral DNA in the blood of each animal throughout the course of the study and all animals scored positive for viral DNA by nested PCR at one or more points following viral exposure (**Fig 1I and 1K,** bottom panels). However, we observed varying effects on the seroconversion of HTLV-1_WT_ to HTLV-1 antigens, measured by Western blot. All animals pretreated with M-T807R1 seroconverted within one month (**Fig 1I and 1L**); seroconversion for those treated with CD8β255R1 antibodies occurred within 1–2 months (**Fig 1J and 1L**). All animals treated with clodronate fully seroconverted within 3 months, but by week 21, 3 out of 5 animals became sero-indeterminate (**Fig 1K**) with low ELISA titers for p24Gag (1:100) that decreased by the third month from viral exposure in these three animals (1:10; **Fig 1L**), suggesting that HTLV-1 replication was not sustained *in vivo*.

**Fig 2:**
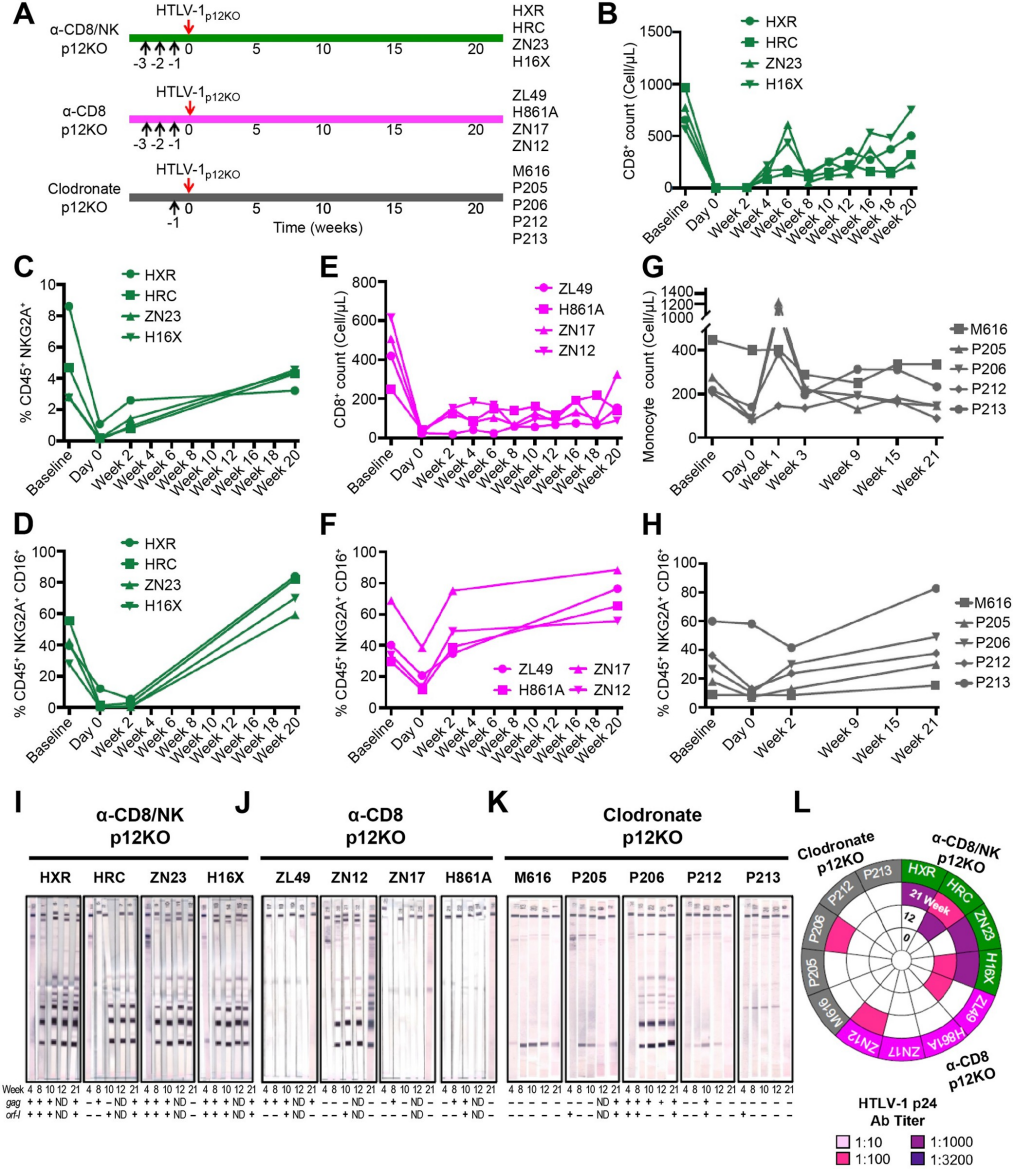
Restoration of HTLV-1_p12KO_ infectivity by double NK/CD8^+^ cell depletion. (**A**) Schematic of study design. Animals in the α-CD8/NK p12KO and α-CD8 p12KO groups were injected intravenously with M-T807R1 and CD8β255R1 respectively at 5mg/kg per day for three days prior to virus inoculation. Macaques in the Clodronate p12KO group were injected with Clodrosome at 20mg/kg one day prior to virus inoculation. Black arrows represent day of treatment with M-T807R1, CD8β255R1, or Clodrosome as indicated, and red arrows indicate inoculation with the lethally irradiated 729.6 lymphoblastoid B-cell lines producing HTLV-1_p12KO_. (**B,E**) Absolute CD8^+^ T-cell numbers shown before, during, and after the administration of M-T807R1 and CD8β255R1 in the (**B**) α-CD8/NK p12KO and (**E**) α-CD8 p12KO groups inoculated with HTLV-1_p12KO_ at baseline, day 0, and after (weeks 2, 4, 6, 8, 10, 12, 16, 18, and 20). (**C**) Frequency of NKG2A^+^ cells identified as Singlets/Live/CD45^+^/CD3^-^CD20^-^/NKG2A^+^ measured before (baseline), during (day 0), and after (weeks 2 and 20) CD8^+^ cell depletion in peripheral blood of the α-CD8/NK p12KO group. (**D,F,H**) Frequency of NKG2A^+^CD16^+^ cells identified as Singlets/Live/CD45^+^/CD3^-^CD20^-^/NKG2A^+^CD16^+^ for the (**D**) α-CD8/NK p12KO, (**F**) α-CD8 p12KO, and (**H**) Clodronate p12KO groups inoculated with HTLV-1_p12KO_ at baseline, day 0, and weeks 2 and 20. (**G**) Absolute monocyte cell numbers in the Clodronate p12KO group before (baseline), during (day 0), and after (weeks 1, 3, 9, 15, and 21) the inoculation with HTLV-1_p12KO_. (**I,J,K**) Sera from the inoculated macaques in (**I**) α-CD8/NK p12KO, (**J**) α-CD8 p12KO, and (**K**) Clodronate p12KO groups tested for reactivity to HTLV-1 antigens during the course of the study (weeks 4, 8, 10, 12, and 21). Animal ID, inoculated viruses, and treatment are indicated above each sample. The week of sera collection is indicated below each western blot strip. Positive amplification of either *gag* or *orf-I* in the blood throughout the course of the study is symbolized by (+); absence of amplification is symbolized by (-). (**L**) HTLV-1 p24Gag antibody titer was measured at weeks 0, 12, and 21. Dilutions of 1:10, 1:100, 1:1000, and 1:3200 were used and color-coded as reported in the figure.

Treatment of macaques with M-T807R1 prior to exposure to HTLV-1_p12KO_ (**Fig 2A**) also resulted in depletion of CD8^+^ cells, NKG2A^+^, and partial depletion of NKG2A^+^CD16^+^ (**Figs 2B–2D** and **S4A**). Following treatment with CD8β255R1 we observed depletion of CD8^+^ cells (**Figs 2E** and **S4B**) and a partial decrease in the NKG2A^+^CD16^+^ subset (**Figs 2F** and **S4B**). Clodronate treatment reduced total monocytes in most animals (**Fig 2G**) and we observed a partial decrease in NKG2A^+^CD16^+^ subset frequency (**Figs 2H** and **S4C**). Strikingly, depletion of both CD8^+^/NK populations restored the infectivity of HTLV-1_p12KO_ in all animals that seroconverted and had antibody titers up to 1:1000 (**Fig 2I and 2L**). In contrast, depletion of CD8^+^ T-cells alone resulted in full seroconversion in only one macaque (animal ZN12) out of four, and ELISA titers remained 1:100 in this animal (**Fig 2J and 2L**). Clodrosome treatment did not result in seroconversion in animals exposed to HTLV-1_p12KO_, except in the case of animal P206, which also had antibody titers of 1:100 (**Fig 2K and 2L**). All animals scored positive by nested PCR for viral DNA in blood at one or more points, except animals ZN12 and M616 treated with the CD8 depleting antibody CD8β255R1 or clodronate, respectively (**Fig 2I–2K**, bottom panels)

In these experiments, we also observed that depleting certain populations of cells affected other cell populations as well. Notably, we observed a consistent decrease in NKG2A^+^CD16^+^ cells in all groups at day 0, but only found a sustained decrease at week 2 after M-T807R1 treatment. Sustained depletion of CD8^+^ cells was observed in animals treated with either anti-CD8/NK or anti-CD8, as expected. Depletion of CD14^+^ and CD16^+^ monocytes at day 0 was found in clodronate treated macaques. However, we did find macaques in all other treatment groups that also experienced moderate decreased CD16^+^ monocyte levels at day 0.

### Occult HTLV-1 infection associated with a pro-inflammatory profile

**Fig 3:**
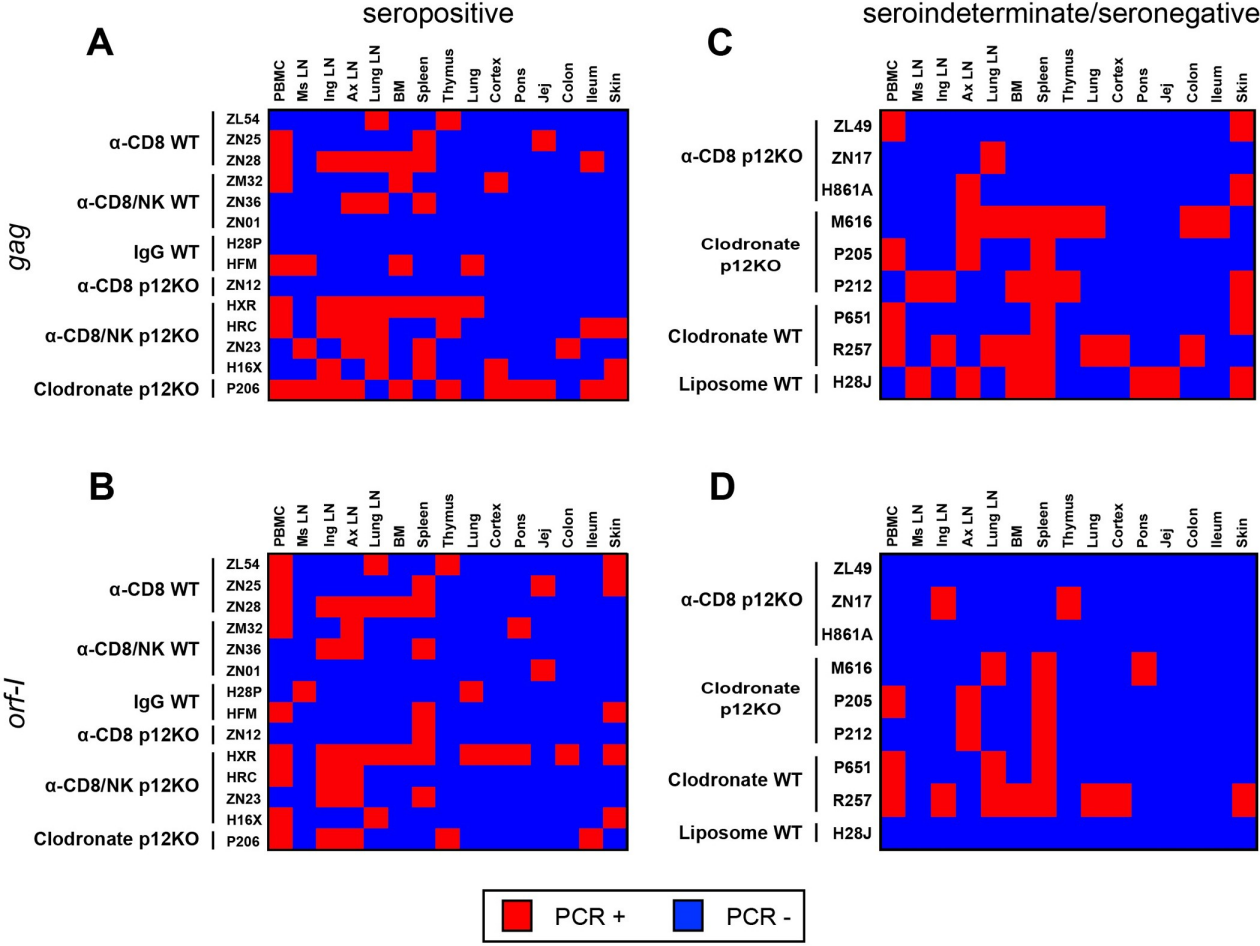
Viral dissemination in infected animals. (**A-D**) Heat map of the nested PCR amplifying the *gag* and *orf-I* genes in biopsies from the ileum, colon, thymus, skin, jejunum, cortex, lung, spleen, inguinal LN, mesenteric LN, axillary LN, and lung LN, collected at the time of euthanasia following virus inoculation, together with PBMCs and bone marrow for (**A,B**) seropositive and (**C,D**) sero-indeterminate/seronegative animals. Genomic DNA was extracted to amplify viral DNA. Nested PCR was performed using primers designed to amplify the *gag* and *orf-I* genes (see Materials and Methods). The *orf-I* PCR products were sequenced to verify HTLV-1_p12KO_ virus versus HTLV-1_WT_. Positive amplification of either (**A,C**) *gag* or (**B,D**) *orf-I* by nested PCR is shown in red; absence of amplification is shown in blue.

Analyses of sero-reactivity to viral antigens revealed three main serological profiles: seropositive, defined as durable serum recognition of four HTLV-1 antigens up to 21 weeks from viral exposure, sero-indeterminate, recognizing fewer than four antigens, and completely seronegative. To determine the virological status of blood and tissues in the exposed animals, we performed DNA PCR for viral DNA in all tissues collected at time of euthanasia at approximately six months from viral exposure from all animals except animals ZJ22, Z151, and HPM treated with clodrosome, and ZJ58 treated with encapsosome (**Tables 2** and **S2**). PBMCs, thymus, bone marrow, spleen, lung parenchyma, ileum, colon, jejunum, cortex, pons, skin, inguinal lymph nodes (LN), mesenteric LN, axillary LN, and lung LN were used as a source of cellular DNA, and primers for the *gag* and *orf-I* genes were used to amplify viral DNA. *Gag* DNA was found in two or more tissues of nearly all seropositive or sero-indeterminate animals (**Figs 3A, 3C,** and **S3A–S3F**). Only animal P213 (treated with clodronate and exposed to HTLV-1_p12KO_) was not included in the analyses since it was both seronegative and had neither *gag* nor *orf-I* DNA in tissues. The *orf-I* DNA was detected less frequently than *gag* in tissues of seropositive animals and was not detected in the mesenteric lymph nodes, colon, or jejunum of sero-indeterminate/seronegative animals (**Fig 3B and 3D**).

**Table 2:** Serological and virological status of animals exposed to HTLV-1_WT_ and HTLV-1_p12KO_.

Treatment	HTLV-1_WT_			HTLV-1_p12KO_		
	α-CD8/NK	α-CD8	Clodrosome/Encapsome	α-CD8/NK	α-CD8	Clodrosome
**Seropositive**	3/3	3/3	1/5**	4/4	1/4	1/5
**Seroindeterminate**	0/3	0/3	4/5	0/4	3/4	4/5
**Seronegative**	0/3	0/3	0/5	0/4	0/4	1/5
**PCR *gag***	+2/3	+3/3	+3/3*	+4/4	+3/4	+4/4
**PCR *orf-I***	+3/3	+3/3	+2/3*	+4/4	+3/4	+4/4
**PCR *gag/orf-I***	+2/3	+3/3	+2/3*	+4/4	+2/4	+4/4

* Only three animals analyzed: P651, R257, H28J.

** Unsustained seroconversion

Animals ZJ22, Z151, and HPM treated with clodrosome, and ZJ58 treated with encapsosome, were not sacrificed and were enrolled in a subsequent study.

**Fig 4:**
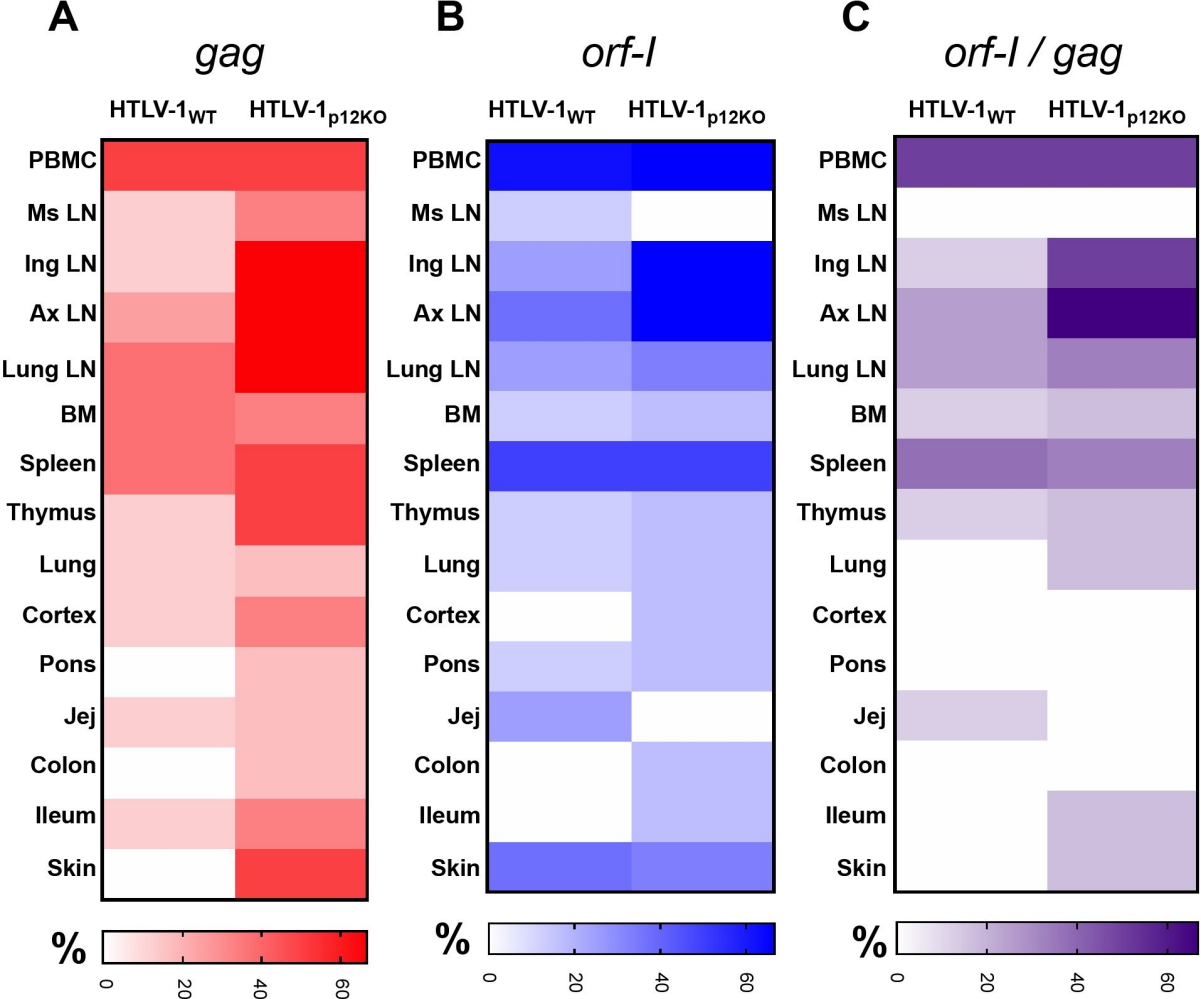
Viral dissemination in infected animals. (**A,B,C**) Heat map charts show the percentage of HTLV-1_WT_ and HTLV-1_p12KO_ inoculated animals with positive amplification for (**A**) *gag* (red), (**B**) *orf-I* (blue), or (**C**) both (purple) are shown for the different tissues.

Next, we investigated the frequency of *gag* and *orf-I* DNA relative to exposure to HTLV-1_WT_ or HTLV-1_p12KO_. Surprisingly, the frequency of tissues carrying *gag* and *orf-I* DNAs did not differ between animals exposed to HTLV-1_p12KO_ or to HTLV-1_WT_ (**Fig 4A and 4B**). Similarly, the overall frequency of tissue positive for both *gag* and *orf-I* DNA did not differ between animals exposed to each virus (**Fig 4C** and **Tables 2** and **S2**).

We next compared the inflammatory profile associated with infection with HTLV-1_p12KO_ to that of HTLV-1_WT_. When we analyzed cytokine profiles in infected animals as separated by treatment, no significant changes were detected. However, this was in part due to the low number of infected animals in some groups. We therefore compared the cytokine profiles associated with infection in HTLV-1_p12KO_ and HTLV-1_WT_ in all animals, regardless of treatment, whose serological and virological status is summarized in **Table 2**. Animal P213 was excluded again from this analysis since it was serologically negative and had no viral DNA in any tissue. When we compared HTLV-1_p12KO_ infected animals at week 21 after exposure when all cell populations have rebounded to baseline, only IL-1β significantly increased. Likewise, when we compared HTLV-1_WT_ infected animals at week 21 after exposure when all cell populations have rebounded to baseline, we detected increases in IL-10 and IL-1β.

Taken together, these results indicate that altering monocyte and NK cellular subsets impact primary HTLV-1 infection and that occult HTLV-1 infection can be established in animals depleted of NK and/or CD8^+^ T-cells or monocytes prior to virus exposure. The data also suggest possible differences in the inflammatory profiles in both acute and chronic infection in animals that became infected by HTLV-1_WT_ or HTLV-1_p12KO_. However, the small number of animals used here precludes definitive conclusions.

### *orf-I* expression inhibits inflammasome activation in primary monocytes and upregulates CD47 “don’t-eat-me” signal in HTLV-1 infected cells

**Fig 5:**
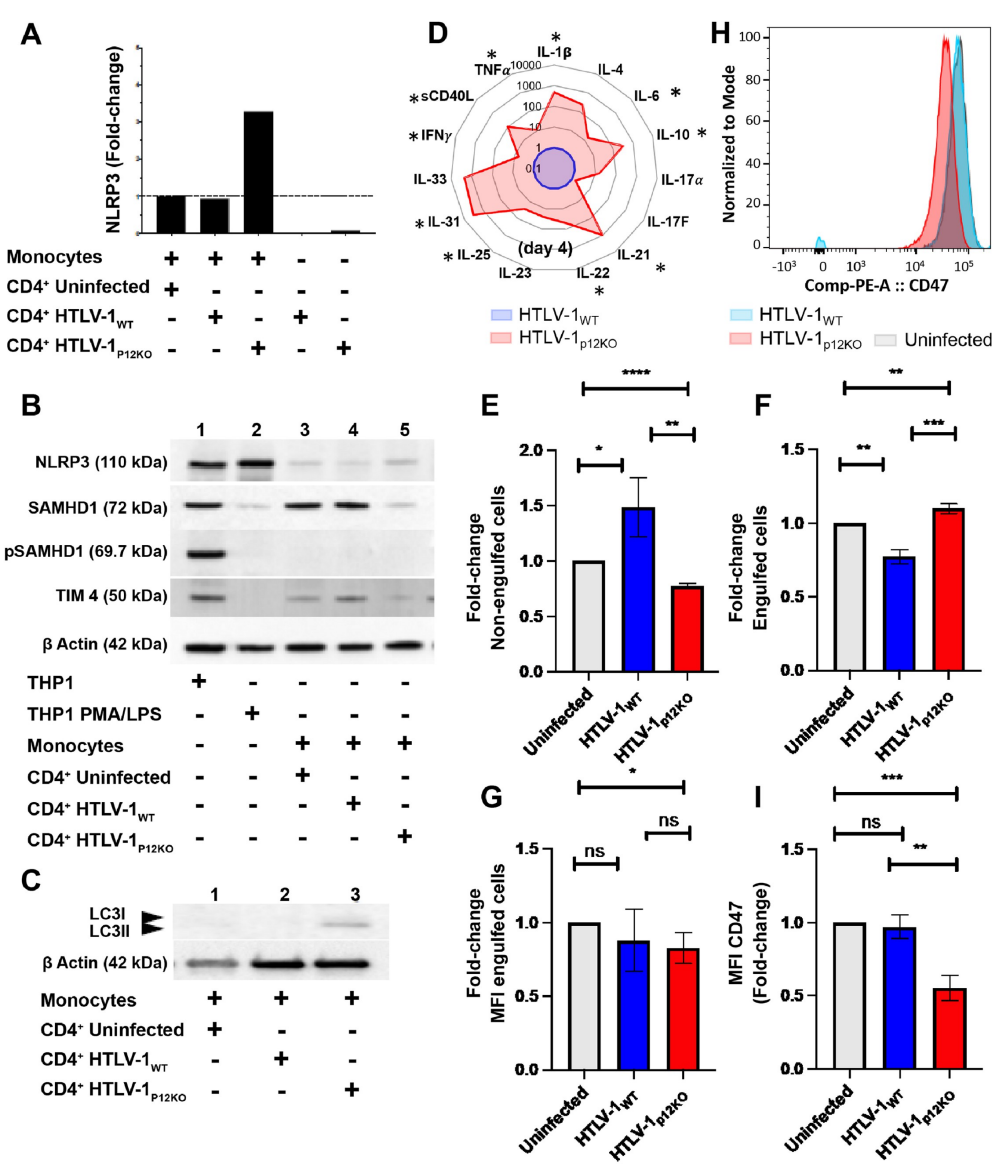
Differences in inflammasome activation and engulfment in monocytes exposed to HTLV-1_p12KO_ and HTLV-1_WT_ infected cells. (**A**) NRLP3 mRNA from cDNA of monocytes co-cultivated with CD4^+^ cells (uninfected), CD4^+^ HTLV-1_WT_, and HTLV-1_p12KO_ was assessed by Real-time PCR. Infected CD4^+^ cells used for the experiment were included in our analysis. Real-time PCR was performed in triplicate and samples were normalized to GAPDH expression. Fold-change was calculated by comparing values with monocytes co-cultivated with uninfected CD4^+^ normalized NLRP3 expression. (**B**) Western blot analyses of NLRP3 (110kDa), SAMHD1 (72kDa), pSAMHD1 (69.7kDa), and TIM4 (50kDa) expression from total cellular extracts of monocytes co-cultivated with uninfected CD4^+^ cells, CD4^+^ HTLV-1 _WT_, and CD4^+^ HTLV-1_p12KO_. THP-1 cells unstimulated and treated with PMA and LPS were included as controls. Protein loading was assessed by ß-actin expression. (**C**) Western blot analyses of LC3I/II (14–18 kDa) expression from total cellular extracts of monocytes co-cultivated with uninfected CD4^+^ cells, CD4^+^ HTLV-1_WT_, and CD4^+^ HTLV-1_p12KO_. (**D**) Spider chart of cytokines and chemokines measured in the cryopreserved supernatants from monocytes isolated from four different donors at three days post-co-cultivation. Cytokine level was analyzed using Bio-Plex Pro Human Th17 Cytokine Panel assays. The following targets were assayed according to the manufacturer’s instructions: IL-1β, IL-4, IL-6, IL-10, IL-17A, IL-17F, IL-21, IL-22, IL-23, IL-25, IL-31, IL-33, IFN-γ, TNF-α, and CD40L. The level of cytokines in the supernatant of HTLV-1_p12KO_ exposed monocytes (isolated from four different donors) was graphed in red as a fold-change compared to the HTLV-1_WT_ (blue). The average is shown in the figure. Asterisks (*) indicate cytokines that were found induced in all donors. Efferocytosis assay of THP-1 cells co-cultivated with HTLV-1 _WT_ or HTLV-1_p12KO_ infected cells or uninfected control cells (**E,F**). The bait cells, THP-1, were labeled with CytoTell Blue. Cells were then seeded in 12 well plates and treated with PMA. THP-1 cells were cultivated for 72 h with effector cells (729.6 cells, 729.6 producing HTLV-1_WT_, or 729.6 producing HTLV-1_p12KO_) previously stained with CFSE and lethally γ-irradiated. A well without effector cells was included for compensation and as a gating control. Fold-change of engulfed cells (CytoTell Blue positive and CFSE positive) and non-engulfed (CytoTell Blue negative and CFSE positive) cells were calculated from 3 independent experiments (see S8C Fig for an example of gating and cellular populations). The HTLV-1 proviral loads (PVL) of 729.6 producing HTLV-1_WT_ and HTLV-1_p12KO_ cells were 776% and 262% respectively. The p19Gag produced in the supernatant 729.6 HTLV-1_WT_ and HTLV-1_p12KO_ in 24 h measured 2832 and 939 pg/ml respectively. (**G**) Mean Fluorescent Intensity (MFI) of engulfed cells. Unpaired t-test was used for statistical evaluation. (**H**) CD47 staining of 729.6 cells, 729.6 HTLV-1_WT_ and 726.9 HTLV-1_p12KO_ cell lines. (**I**) Fold-change of MFI CD47 was calculated from 3 independent experiments. Unpaired t-test was used for statistical evaluation.

Prior work has demonstrated that expression of *orf-I* is essential for sustained persistence of viral DNA in monocyte-derived dendritic cells and the monocytic cell line THP-1 [38,44]. In particular, we observed that the p8 protein is essential for the detection of viral DNA in the THP-1 monocyte cell line infected via cell-free virus [38]. To further assess the impact of *orf-I* expression on monocyte functionality, we used *in vitro* adherent human primary monocytes purified from the blood of HTLV-1 negative individuals. Although monocytes and dendritic cells can be infected with cell-free virus, the primary route of viral infection is thought to still occur through cell-to-cell contact. We therefore examined HTLV-1 infection through co-culture. Monocytes and irradiated MHC-I-matched CD4 cells infected with either HTLV-1_WT_ or HTLV-1_p12KO_ were co-cultivated for 24 hours and extensively washed (day 1) to eliminate CD4^+^ cells. Input CD4 producing cells were normalized for virus production by supernatant p19Gag levels (**S6A Fig**). For some co-cultures equivalent p19Gag resulted in different input in viral DNA (**S6B Fig**). Co-culture supernatants were harvested at days 2, 3, and 4. Even though p19Gag release in the supernatant at day 2 was higher in monocytes exposed to HTLV-1_p12KO_ than to HTLV-1_WT_, p19Gag production decreased in all cell cultures (**S6C and S6D Fig**). Invariably, following exposure to HTLV-1_p12KO_ we observed a pronounced decrease in the percentage of CD14^+^CD16^+^ intermediate and CD14^-^CD16^+^ non-classical subsets, particularly in HTLV-1_p12KO_ co-cultures (**S6F and S6G Fig**). We hypothesized that exposure of monocytes to the irradiated HTLV-1_p12KO_ infected cells may result in more pronounced inflammasome activation, an effect that may be mitigated by the expression of *orf-I* in HTLV-1_WT_. This hypothesis was supported by the finding in monocytes of increased expression of NLRP3, a component of the inflammasome, by RT-PCR at the mRNA level, following exposure to HTLV-1_p12KO_ (**Fig 5A**). Only a marginal difference or no difference in the NLRP3 protein levels were detected. The restriction factor SAMHD1, shown to limit HTLV-1 infection of monocytes *in vitro* [74], was reduced in HTLV-1_p12KO_ co-cultured monocytes compared to those cultured with uninfected CD4 cells or HTLV-1_WT_ producing cells (**Fig 5B,** compare lane 5 to lanes 3 and 4). This is consistent with increased inflammasome activation in HTLV-1_p12KO_ monocyte cultures [75]. In addition, we did not observe phosphorylation of SAMHD1, indicating that our cultures were activated and differentiated [76]. The monocytic cell line THP-1 and PMA/LPS differentiated THP-1 cells were used as antibody controls. We also noted that the lipidated form of LC3 (LC3-II) was higher in monocytes exposed to HTLV-1_p12KO_, a finding consistent with a feedback activation of autophagy [77] (**Fig 5C,** lane 3). Interestingly, the expression of T-cell immunoglobulin and mucin domain containing 4 protein (TIM-4), a receptor that recognizes phosphatidylserine on apoptotic cells (pdTS) and is essential in early monocyte engulfment of apoptotic cells during efferocytosis [78], was also higher in cells infected with HTLV-1_WT_ (**Fig 5B,** lane 4). To demonstrate functional activation of the inflammasome, we measured cytokines in the supernatants of MHC-I-matched CD4^+^ (HTLV-1 infected or uninfected) monocyte co-cultures from four healthy individuals. We observed an increase in the release of several cytokines, including the inflammasome-dependent cytokines IL- 31 and IL-1β, in cells exposed to HTLV-1_p12KO_ (**Fig 5D**).

**Fig 6:**
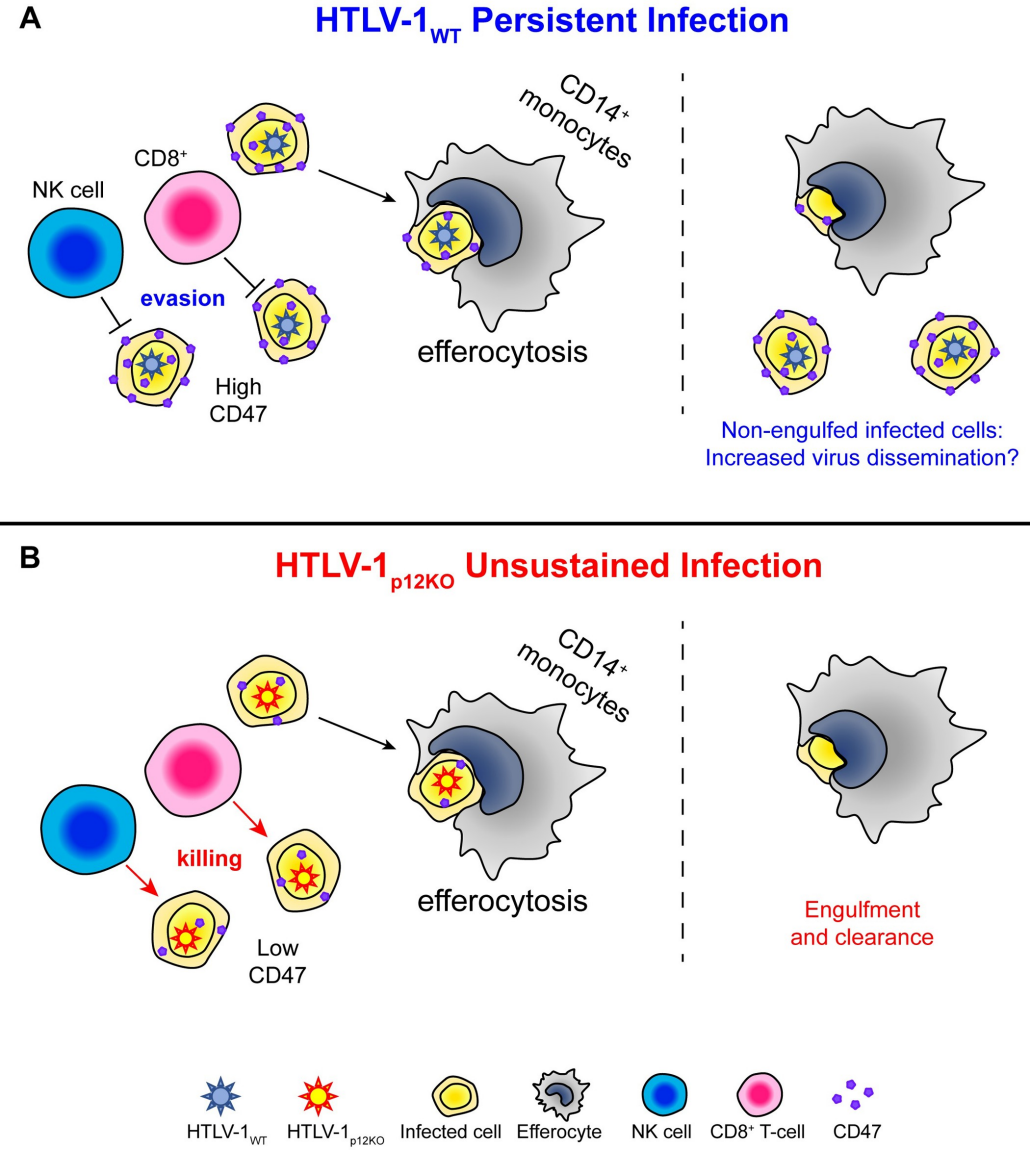
Role of *orf-I* gene and immune cells in primary HTLV-1 infection. (**A**) Graphical representation of how HTLV-1_WT_ infected cells escape immune recognition by counteracting cytocidal NK and CTL activity via downregulation of ICAM-1, ICAM-2 [49], and MHC- I [56] and partly elude efferocytosis via *orf*-*I* upregulation of the “don’t-eat-me” CD47 molecule as demonstrated in the current work. (**B**) The absence of *orf-I* expression would result in lower CD47 expression on the surface of HTLV-1_p12KO_ infected cells and susceptibility of infected cells to both NK cells and CTLs as well as to efferocytosis, explaining the inability of this mutant virus to persist in the host.

The decline of p19Gag production in CD4^+^ cell/monocyte co-culture observed in our experiments (**S6C and S6D Fig**) raised the possibility that monocytes may not be infected with canonical viral integration in the host genome but may rather engulf apoptotic infected CD4^+^ cells. Indeed, confocal microscopy of CD4^+^ infected T-cells (stained with CellTracker Blue) and monocytes stained to label the plasma membrane (WGA594 in red) prior to co-cultivation demonstrated clearly that primary human monocytes engulf HTLV-1 infected cells (**S7A Fig**). We next performed high-throughput sequencing (HTS) and compared viral integration sites in the input CD4^+^ cells with their matched monocyte co-cultures at day 4 (following removal by extensive washing of the CD4^+^ T-cells). These analyses revealed the relative abundance of unique integration sites (**S7B Fig**) and demonstrated that a high number of sites are identical in monocytes and CD4^+^ T-cells (13.5% and 5.3% of all integration sites observed in WT and p12KO cells, respectively), suggesting that monocyte engulfment is the predominant source of both p19Gag in the supernatant and of viral DNA in monocytes. We also observed a minority of integration sites that appeared to be unique to monocytes. However, because the infected CD4^+^ T-cells are a mixed population of cells and not clonal, we cannot exclude the possibility that monocytes had engulfed rare CD4^+^ infected T-cells and that viral DNA arises from “passenger T-cells” rather than from integration in the monocyte genome. Our data therefore leave the question of HTLV-1 integration in the monocytes’ genome unresolved. Similar results were obtained by co-culturing MHC-I unmatched CD4^+^ T-cells and monocytes (**S7C Fig**). We extended our analysis to macaque primary monocytes, and we confirmed engulfment of lethally irradiated HTLV-1 infected cells in the monocytes of the four macaques (**S8A and S8B Fig**). Since an essential function of monocytes is efferocytosis, a highly regulated clearance of apoptotic cells that is hijacked by several pathogens [79], we next investigated whether *orf-I* expression affected THP-1 engulfment of HTLV-1 infected cells. We observed that uninfected and HTLV-1_p12KO_ infected cells were engulfed more efficiently than HTLV-1_WT_ infected cells, leaving a larger proportion of HTLV-1_WT_ infected cells non-engulfed (**Figs 5E, 5F, and S8C**). The MFI of engulfed cells did not differ between those infected by HTLV-1_WT_ or HTLV-1_p12KO_ (**Fig 5G**). The difference between HTLV-1_WT_ and HTLV-1_p12KO_ infected cell engulfment was not due to differences in their level of apoptosis before or after irradiation (72 hours) at the time of readout of the efferocytosis assay (**S8D Fig**). Rather, we observed that the level of expression of the CD47 “don’t-eat-me” signal was significantly higher in HTLV-1_WT_ than HTLV-1_p12KO_ infected cells (**Fig 5H and 5I**), suggesting that HTLV-1_p12KO_ infected cells are more susceptible to engulfment by efferocytes, likely because of lower surface expression of the CD47 molecule (**Fig 6**). Whether or not *orf-I* expression also decreases cargo destruction by inhibiting inflammasome activation and creating a “trojan horse” mechanism of viral transmission will require further investigation.

## Discussion

Chronic HTLV-1 infection is associated with unrelenting immune activation accompanied by high levels of both pro- and anti-inflammatory cytokines [80,81]. This outcome likely results from the host’s continuous, albeit unsuccessful, attempts to eradicate viral infection and maintain tissue homeostasis [82]. HTLV-1 establishes a life-long virus reservoir, and viral DNA has also been found in other cell types, such as neutrophils, epithelial cells, and monocytes [12,14,83], though there is no compelling evidence that viral integration (provirus) occurs in these cell types. Instead, our data in monocytes suggest that infected cell engulfment may be a predominant mechanism of viral invasion, and this may also be the case for neutrophils or epithelial cells, both of which are able to engulf and eliminate senescent or apoptotic cells [84].

In contrast, infection with viral integration occurs in memory CD4^+^ and CD8^+^ T-cells [85]. Upon antigen stimulation, however, memory cells carrying HTLV-1 can express Tax and produce virus with an increased risk of host immune recognition [86,87]. While the mode of HTLV-1 transmission may have evolved to spare free virus from antibody recognition, there remain several obstacles for the survival of infected cells, including natural killer cells, phagocytic monocytes, and CTL.

*In vitro* studies have demonstrated that HTLV-1 has evolved strategies to escape immune recognition. Viral proteins such as p13 and p30 encoded by *orf-II* are able to modulate interferon responses [32,88–90], and p8 and p12 encoded by *orf-I* counteract NK and CTL functions [38,48,49,52,56]. *In vivo*, both *orf-I* and *orf-II* ablation by single point mutations of HTLV-1 molecular clones resulted in loss of virus infectivity [44]. Remarkably, these studies demonstrated that reversion of the single point mutation to wild type in replete condition occurred only with the HTLV-1 molecular clone ablated in *orf-II*, suggesting that sufficient rounds of replication still occurred in macaques to allow for the selection of a revertant virus, whereas reversion of HTLV-1_p12KO_ mutation to wild type was not observed in replete macaques [44]. Of interest, however, is that HTLV-1_p12KO_ reversion to wild type did occur in humanized mice [91], perhaps because the incomplete reconstitution of the human immune system in this murine model allowed sufficient rounds of HTLV-1_p12KO_ replication to select for viral fitness. No significant reconstitution of human monocytes was detected in these humanized infected mice, suggesting a possible role for monocytes in the early phase of HTLV-1 infection [91].

Here, we used the macaque model of HTLV-1 infection to directly address the importance of monocytes and NK and CD8^+^ cells. The macaque model has some limitations however, as the HTLV-1_WT_ virus that we use infects approximately only half of the animals, and the virus load is lower than in HTLV-1 infected carriers. Thus, to increase our chances of understanding the role of these immune cells in primary infection, we designed experiments using both HTLV-1_WT_ and HTLV-1_p12KO_, capitalizing on the lack of infectivity of the HTLV-1_p12KO_ molecular clone. We report here that depletion of NK cells is associated with exacerbation of HTLV-1_WT_ infectivity and restoration of HTLV-1_p12KO_ infectivity, highlighting the critical role of *orf-I* expression *in vivo*. The finding that HTLV-1_WT_ infection is also exacerbated by NK depletion further supports this finding and suggests that the stoichiometry of *orf-I* expression likely differs among infected cells. Ablation of *orf-I* expression in HTLV-1_p12KO_ resulted in inflammasome activation and increased lipidation of LC3, a protein that promotes phagolysosome assembly and suppresses proinflammatory signaling [92] in primary human monocytes. In addition, surface expression of the CD47 “don’t-eat-me” molecule is higher on HTLV-1_WT_ than HTLV-1_p12KO_ infected cells, resulting in less effective monocyte engulfment of cells infected by wild type virus. Thus, our data suggested the hypothesis that HTLV-1_WT_ infected cells may escape immune recognition and transiently elude efferocytic function of monocytes via upregulation of the CD47 molecule by *orf-I* and spare HTLV-1 infected cells (**Fig 6**).

Prior works provide support for a possible role of viral proteins in the phagocytic function of monocytes. The *orf-I* proteins both increase cytoplasmic calcium [58,59], which promotes phagolysosome formation [93], and bind to the 16KA subunit of vacuolar ATPase, an essential enzyme regulating acidification of lysosomes for recycling cellular components during efferocytosis [94,95]. In addition, p8 binds to the vasodilator-stimulator phosphoprotein (VASP) that promotes actin filament elongation, a key step in phagocytosis [62,63], and the p30 protein encoded by *orf-II* promotes the expression of the pro-resolving IL-10 cytokines [32] associated with efferocytosis.

The overall picture that emerges suggests the hypothesis that HTLV-1 hijacks efferocytosis and decreases the engulfment of virus-infected T-cells via *orf-I* upregulation of surface expression of the CD47 “don’t-eat-me” molecule. This effect likely complements *orf*’s ability to downregulate ICAM-1 and ICAM-2, and MHC-I, which respectively confer infected cells with resistance to cytocidal NK and CTL activity (**Fig 6A**). The possibility of *orf-I* expression also transiently protecting engulfed cells from degradation and further facilitating the spread of virus by migratory efferocytes to tissues is only hypothetical at present and has not been investigated here. However, defective efferocytosis could create a durable and vicious inflammatory response unable to clear the virus by inducing further inflammation [96] and Treg cell differentiation via production of IL-10 and TGF-β.

The increased expression of these cytokines and Treg counts are both hallmarks of HTLV-1 infection and are thought to contribute to HTLV-1 pathogenesis [82]. The possibility that defective efferocytosis may play a role in maintaining persistent viral infection is compatible with the abortive infection observed with depletion of monocytes/macrophages in animals prior to exposure to HTLV-1_WT_, despite the accelerated but transient seroconversion and the lack of infectivity of HTLV-1_p12KO_ in the same conditions. The transient decrease of monocytes induced by clodronate in the first few days following exposure may further spare infected cells from efferocytosis, expanding the early pool of infected T-cells with a resultant increase in viral antigens and enhanced sero-conversion. In turn, however, increased antigen exposure could increase the activation of cytotoxic NK and CD8 cells, impairing viral persistence and resulting in unsustained seroconversion. An alternate hypothesis is that clodronate affects the establishment of an early viral reservoir in monocytes necessary to maintain viral persistence. Additional experiments with simultaneous *in vivo* depletion of NK, CD8, and monocytes will be necessary to address these hypotheses.

HTLV-1 infection has been reported to significantly alter monocyte subsets, with chronic infection resulting in lower frequency of classical monocytes and increased intermediate and non-classical monocytes [12]. Further work will be needed to mechanistically assess which of the tightly regulated steps of efferocytosis are regulated by the non-structural HTLV-1 proteins in virus infected cells and in monocytes. This work will be essential to identify possible druggable targets to ameliorate clearance of virus-infected cells and decrease the immune activation observed in HTLV-1 infection [81,84,97].

The frequency and function of NK cells is also altered in HTLV-1 infection [98]. NK cells undergo spontaneous proliferation in infected individuals and their frequency correlates with their viral DNA burden [99]. An anecdotal case wherein passive transfer of amplified natural killer (ANK) cells to a patient with smoldering adult T-cell leukemia resulted in complete remission [100] also suggests the importance of NK cells in chronic HTLV-1 infection. Little is known however on the role of NK cells in primary infection. We show here by NK depletion in animals prior to virus exposure that NK cells are key to restricting HTLV-1 primary infection. However, it remains unclear whether the protective role of NK cells in primary infection is conveyed via their interaction with monocytes/macrophages, dendritic cells, T-cells, and/or B cells to shape innate and adaptive immunity, or via their innate cytocidal function.

In summary, our data are consistent with a critical role of HTLV-1 infected cell engulfment by monocytes for productive and persistent viral infection and suggest that continuous monocyte engagement by virus infected cells may underlie the pro-inflammatory profile observed in HTLV-1 infected individuals. Hence, preventive vaccines will need to induce not only adaptive, but also innate responses able to promptly eliminate virus infected cells.

## Materials and methods

### Ethics statement

The animals used in this study were colony-bred Indian rhesus macaques (Macaca mulatta) obtained from either Alpha Genesis Inc. (Yemasee, SC), Primate Products Inc. (Immokalee, FL), the National Institute of Child Health and Human Development (NICHD, Rockville, MD), or Covance Research Products (Princeton, NJ). Animals were housed at the National Institutes of Health, Bethesda, MD (Protocol VB033). Animals were cared for in accordance with Association for Assessment and Accreditation of Laboratory Animal Care (AAALAC) standards in an AAALAC-accredited facility (OLAW, Animal Welfare Assurance A4149-01). All animal care and procedures were carried out under protocols approved by the NCI and/or NIAID Animal Care and Use Committees (ACUC; Protocol number: VB033). Animals were closely monitored daily for any signs of illness, and appropriate medical care was provided as needed. Animals were socially housed per the approved ACUC protocol and social compatibility except during the viral challenge phase when they were individually housed. All clinical procedures, including biopsy collection, administration of anesthetics and analgesics, and euthanasia, were carried out under the direction of a laboratory animal veterinarian. Steps were taken to ensure the welfare of the animals and minimize discomfort of all animals used in this study. Animals were fed daily with a fresh diet of primate biscuits, fruit, peanuts, and other food items to maintain body weight or normal growth. Animals were monitored for mental health and provided with physical enrichment including sanitized toys, destructible enrichment (cardboard and other paper products), and audio and visual stimulation.

### Generation and Characterization of HTLV-1 infected human primary CD4^+^ T-cells

Primary CD4^+^ T-cells were isolated from PBMCs from healthy donors using negative-selection beads (StemCell, Cambridge, MA). Stable HTLV-1-producing CD4^+^ T-cell lines were established by co-cultivation of uninfected donor primary CD4^+^ T-cells with γ-lethally irradiated 729.6 human lymphoblastoid B-cell lines producing HTLV-1_WT_ or the p12 viral mutant HTLV-1_p12KO_ [38,44]. CD4^+^ T-cells were maintained in culture for several months in RPMI-1640 medium supplemented with 20% FBS and 100 U of IL-2. HTLV-1 infection was determined by p19Gag antigen detection by a commercially available ELISA in the culture supernatants of 10^6^ cells washed and seeded in a 24-well plate in 1 mL complete RPMI (Zeptometrix, Buffalo, NY). According to the manufacturer, sensitivity of detection is 25 pg/mL. Viral genomic sequences were verified by sequencing the ClaI-SalI fragment of the pAB molecular clone. Viral DNA load was determined for each established CD4^+^ cell culture. Briefly, genomic DNA was extracted from infected CD4^+^ T-cells (DNeasy Blood and Tissue Kit; Qiagen, Germantown, MD) and 50 ng of DNA were used in Real-time PCR analysis of HTLV-1 (*tax*) [44]. The *RNase P* gene, detected with the TaqMan RNase P control reagents kit (Applied Biosystems, Foster City, CA), was used as the endogenous reference. HTLV-1 viral DNA levels were calculated by the following formula: copies of HTLV-1 (*pX*)/(copies *RNase P*)×100 cells.

To assess surface markers, HTLV-1 infected CD4 cells were stained with anti-CD3-Alexa Fluor 700, anti-CD4-PerCP-Cy5.5, anti-CD8-Brilliant Violet 650 (BV650), and anti-CD19-BV605 antibodies (BD Biosciences, San Jose, CA), and with Live/Dead fixable aqua dead cell stain (Thermo Fisher Scientific, Eugene, OR). Samples were acquired on an LSRII flow cytometer using FACSDiva 8.0 software (BD Biosciences) and analyzed using FlowJo (BD Biosciences).

### Co-Cultivation of HTLV-1 infected human primary CD4^+^ T-cells and primary monocytes

Primary monocytes were obtained from heparinized human peripheral blood from healthy donors and were treated with Ficoll-Paque plus (GE Healthcare, Chalfont St. Giles, United Kingdom) according to the manufacturer’s instructions. Monocytes were separated using EasySEP Human Monocytes Enrichment Cocktail without CD16 depletion (StemCell) as described by the manufacturer, checked for purity by flow cytometry, and cultured in DMEM 1X (GIBCO, Thermo Fisher Scientific) with 20% FBS and 10% of Human AB Serum (Mediatech, Inc. Manassas, VA). Monocytes were co-cultivated with lethally γ-irradiated primary CD4^+^ T-cells (matched and unmatched donors) infected with HTLV-1_WT_ or HTLV-1_p12KO_, or uninfected primary CD4^+^ T-cells used as negative control. The number of infected CD4^+^ T-cells used in the co-cultures was normalized based on the level of p19Gag in the supernatant (Zeptometrix) and the Viral DNA load.

Twenty-four hours after co-cultivation, monocytes were washed and maintained in DMEM supplemented with 10% FBS and 10% human AB sera for 72 h. The supernatants were collected for p19Gag measurement, and the media was changed daily. Three days post co-cultivation, monocytes were harvested, and flow cytometry analyses were performed to evaluate the purity of the culture and the monocyte phenotype. Cells were stained with CD14, CD16, CD3, C4, and CD20 antibodies (BD Biosciences), and Live/Dead aqua viability dyes (Thermo Fisher Scientific).

Total RNA was extracted from the monocyte culture 72 h post co-cultivation using RNeasy RNA isolation kit (Qiagen) as per the manufacturer’s instructions. All RNA samples were dissolved in 50 μl of nuclease-free H_2_O and quantified using a Nanodrop ND-2000 apparatus (Thermo Fisher Scientific). Reverse transcription was performed on 200 ng of total RNA using QuantiTect Reverse transcription Kit according to the manufacturer’s instructions (Qiagen). Real-time PCR was then performed in triplicates using SYBR Green PCR Master Mix (Applied Biosystems) and specific primers for NLRP3: NLRP3-F, 5’- AGCTGCCTCCTGCAGAACCT -3’and NLRP3-R, 5’-GGTCAGCTCAGGCTTTTCTTCT -3’. The qPCR was performed as follows: denaturation at 95° C for 5 min, followed by amplification with 40 cycles of 95° C for 10 s, followed by 60° C for 30 s. The data were normalized to GAPDH, and the fold-change was calculated as 2-ΔΔCt, where ΔΔCt represents the Ct (sample)—Ct (control).

### Animal inoculation and treatments

Thirty-one rhesus macaques uninfected by SIV/SHIV as demonstrated by several consecutive negative PCR were randomized into nine groups based on sex and their prior enrollment in other studies (**S1 Table**). Animals were treated for three days with one of three antibodies: either one of the two different depleting anti-CD8 monoclonal antibodies (the clone M-T807R1 that targets the α/α chain or the CD8β255R1 targeting the α/β chain), or with the isotype control antibody IgG OKT3 reactive against the human CD3 molecule used as control. The three different antibodies were purchased from the NHP Reagent Resource Program (University of Massachusetts Medical School, Worcester, MA) and were injected intravenously at 5mg/kg per day for three days prior to virus inoculation. Animals were then inoculated intravenously with the lethally γ-irradiated 729.6 B cells producing equivalent levels of p19Gag antigen and expressing a similar viral DNA load from the HTLV-1_WT_ and HTLV-1_p12KO_ or 1x10^8^ of the parental uninfected 729.6 cells used as a control [38,44]. In order to assess the role of the monocytes during the acute HTLV-1 infection in macaques, animals were treated intravenously once with 5mg/kg of either Clodrosome or Encapsome (Encapsula NanoSciences LLC, Brentwood, Tennessee) one day prior to the inoculation of irradiated HTLV-1_WT_ or HTLV-1_p12KO_ producing cells (**Table 1**). The number of virus infected cells injected was normalized for p19Gag production and viral DNA level measured as above. Animals were observed for more than 20 weeks and then euthanized to study viral dissemination in tissues.

### HTLV serology and viral DNA detection

Reactivity to specific viral antigens in the plasma of infected animals were detected with the use of a commercial HTLV-1 western immunoblot assay (GeneLabs Diagnostics, Redwood City, CA). Genomic DNA from the PBMC, bone marrow, and biopsies from ileum, colon, thymus, skin, jejunum, cortex, lung, spleen, and inguinal LN, mesenteric LN, axillary LN, and lung LN were isolated from animals at 21 or 31 weeks (euthanasia) post-inoculation using the DNeasy Blood and Tissue Kit (Qiagen). One hundred nanograms of DNA were used as templates for the first round of PCR amplification using primers gag-F1, 5′-GGCCAAATCCTTTCCCGTAG-3′ and gag-R1, 5′-GTTGTGGATTGTTGGCTTGG-3′ or p12-F1, 5′-CCTCGCCCTTCCAACTGTCT-3’ and p30-R1, 5′-AGGAAGGAGGGTGGAATGTT-3’. Three microliters of the PCR reaction were used as a template for nested PCR using primers gag-F2, 5′-GTCCCTCCAGTTACGATTTCC-3′ and gag-R2, 5′-AGGGAGGAGCAAAGGTACTG-3′ or p12-F2, 5’-CGCCTTCCAACTGTCTAGTATAGC-3’and p30-R2, 5’-GGGAGTCGAGGGATAAGGAA-3’. The PCR conditions used were 94° C for 2 min, followed by 35 cycles of 94° C for 30 s, 55° C for 30 s, 68° C for 60 s, and a final extension at 68° C for 7 min, and a hold at 4° C. Platinum High Fidelity PCR SuperMix (Invitrogen, Carlsbad, CA) was used according to the manufacturer’s protocol. Correctly sized amplicons were identified by 1% agarose gel electrophoresis. Sanger sequencing was carried out on the *orf-I* amplicons at the Center for Cancer Research Genomics Core at the National Cancer Institute, NIH.

### HTLV-1 p24 antibody titer

HTLV-1 p24 antibodies in plasma samples from macaques were detected and quantified against purified HTLV-1 p24 protein using an ELISA assay (Advanced BioScience Laboratories, Inc., Rockville, MD). Each well of the ELISA plate was coated with HTLV-1 p24 and incubated overnight at 4°C. Wells were then emptied and blocked with PBS for 1 h at room temperature. Serially diluted samples were added to the wells and incubated for 1 h at 37°C. The plate was then washed with PBS Tween 20 and incubated for 1 h at 37°C with the diluted goat anti-human IgG HRP (Kirkegaard & Perry Lab Inc., Gaithersburg, MD). The plate was washed and the K-Blue Aqueous substrate (Neogen, Lansing, MI) was added to the wells and incubated for 30 min at room temperature. The reaction was stopped by adding H_2_SO_4_ and the plate was Read at 450 nm (E-max reader, Molecular Devices, San Jose, CA).

### Western blot

Whole cell extracts were prepared and 15 μg samples were boiled for 5 min at 100°C with 2x sample buffer with 10% β-mercaptoethanol. The denatured proteins were separated by SDS-PAGE (NuPAGE 4–12% Bis-Tris Protein Gels, Thermo Fisher Scientific) for approximately 2 h at 100 A and transferred to a 7.0 cm x 8.4 cm, 0.45 μm pore size, hydrophobic PVDF (Immobilon-P PVDF, Millipore Sigma, St. Louis, MO), previously activated with methanol for 1 min. Proteins were transferred for 1 h 30 min at 140 mA. The membranes were incubated overnight at 4°C with primary antibodies to NLRP3 (Rabbit D4D8T, Cell Signaling Technology, Danvers, MA), SAMHDI (Mouse ab67820, Abcam, Cambridge, MA), and Phospho-SAMHD1 (Rabbit D7O2M, Cell Signaling Technology), LC3A/B (Rabbit D3U4C, Cell Signaling Technology) in PBS containing 0.1% Tween 20 and 0.25% milk. Membranes were washed in PBS 0.1% Tween and exposed to a horseradish peroxidase-conjugated goat secondary anti-Mouse or anti-Rabbit antibody (1:10,000; ab112767, Abcam). Proteins were visualized by chemiluminescence using a ChemiDoc Imaging System (Bio-Rad Laboratories, Hercules, CA). Densitometric analysis was performed using Image Lab Software.

### High-throughput sequencing (HTS) method to map viral integration sites

To explore HTLV-1 clonality in monocytes following co-cultivation with infected CD4^+^ T-cells, we used an optimized HTS method to map proviral integration sites in the human genome and to simultaneously measure the abundance of the corresponding clones, as previously described [91,101]. Pie charts in the figure illustrate the relative abundance of HTLV-1_WT_ and HTLV_p12KO_ provirus in monocytes following co-cultivation of matched and unmatched infected CD4^+^ T-cells. Each slice represents a unique integration site and size corresponds to the relative abundance.

### Multiplex assay of monocyte supernatants and plasma collected from rhesus macaques

Cryopreserved supernatants from monocyte infected cultures (1 and 3 days co-cultivation) were analyzed using Bio-Plex Pro Human Th17 Cytokine Panel assays (Bio-Rad Laboratories). The following targets were assayed following the manufacturer’s instructions: IL-1β, IL-4, IL-6, IL-10, IL-17A, IL-17F, IL-21, IL-22, IL-23, IL-25, IL-31, IL-33, IFN-γ, TNF-α, and CD40L.

Cryopreserved plasma collected from rhesus macaques at baseline, 24 h, at 2, 4, and 8 weeks after inoculation of the virus, and at euthanasia were analyzed using MILLIPLEX Non-Human Primate Multiplex assays (EMD Millipore, Billerica, MA). The following targets were assayed following the manufacturer’s instructions: IL-18, IL-12/23, TNF-α, IL-8, IL-6, IL-10, IL-1β. After thawing the plasma on ice, 25 μl of each were briefly loaded into the well and mixed with 25 μl of assay buffer and 25 μl magnetic beads. The plates were incubated under agitation at 4° C for 18 h. After washing, 25 μl of detection antibody were added to each well.

### Flow cytometry analysis of rhesus macaque samples

Two staining panels were developed for analysis of macaque PBMC from these experiments. A 22-color panel was designed to examine T-cell and NK cell responses to treatment (CD2, CD3, CD4, CD7, CD8 [2 clones], CD14, CD16, CD20, CD56, NKp44, NKG2A, CD45, CD25, CD62L, CD28, CD95, CCR4, CD107a, CD127, and HLA-DR). A 16-color panel was designed to measure monocyte responses to treatment (CD3, CD4, CD11b, CD14, CD15, CD16, CD20, CD33, CD45, CD68, CD86, CCR2, CXCR4, PDL1, and HLA-DR). All antibodies were selected based on cross-reactivity with rhesus macaques and fluorochrome availability. Antibody information for both panels, including clones and fluorochromes, is listed in **S3 Table**. Frozen PBMCs were thawed, counted, and stained for flow cytometry analysis. Live/Dead Fixable aqua dye (Thermo Fisher Scientific) was included in each panel to eliminate dead cells.

Briefly, frozen PBMCs were thawed, counted, and resuspended at 1x10^7^ cells/ml in D-PBS without Ca^2+^ or Mg^2+^ (Thermo Fisher Scientific). Cells were aliquoted into three 12 x 75 mm polystyrene tubes, 100 μl per tube, (Unstained, 22-color panel, 16-color panel) and stained for 30 min on ice. Samples were washed with D-PBS and resuspended in 1% ultrapure formaldehyde (Tousimis, Rockville, MD). Samples were acquired immediately on a BD FACSymphony A5 analyzer using FACSDiva 8 software. Data were analyzed using FlowJo Version 10.6.

### Immunofluorescence

Primary monocytes (1x10^5^) were seeded in an uncoated μ-well (Ibidi, Fitchburg, WI). Prior to co-cultivation, 48x10^3^ CD4^+^ infected cells (HTLV-1_WT_ and HTLV_p12KO_) were labeled with 1 μM of Cell Tracker Blue CMAC (7-amino-4-chloromethylcoumarin, 25 μM) for at least 30 min before they were washed once with saline and incubated with medium overnight. Cells were washed once with PBS before their addition to the μ-well containing monocytes. The cell membrane of primary monocytes was pre-stained with 1.67μg/ml wheat germ agglutinin (WGA)-Alexa Fluor 594 in medium (8 min at 37°C), followed by two gentle washes with saline or PBS, always leaving 100 μl in the well prior to the addition of CTB-stained CD4^+^ infected cells. 24 h after co-culturing, the cells were fixed with 4% PFA for 15 min at room temperature followed by two washes with PBS. Cells were investigated with LSM 510 confocal microscope with alpha Plan-Apochromat 63x/1.40 oil objective using the following laser sets: Argon (Ar) - 30mW/488 and Helium/Neon I (HeNeI) - 1mW/543 using Plan-Apochromat 63X, 1.40 NA Zeiss objective and GaASP detectors (Alexa 594) and PMT (DAPI). Forty-two Z-stack images of 0.45 μm thickness were acquired with pixel size of 264 nm at 12-bit image depth with averaging (setting 2) and pixel dwell time of 1.27 ms in the following channels: Red (Ex 594 nm, 10% of laser power; Em 597–695 nm) and Blue (Ex 405 nm, 1% of laser power; Em 410–470 nm). Figures were made using the orthogonal view option in the Fiji software [102].

### Efferocytosis assay

The THP-1 human monocytic cell line or primary monocytes were labeled with CytoTell Blue (Cayman Chemical, Ann Arbor, MI) or CellTrace Far Red Cell (ThermoFisher Scientific, Eugene, OR) according to the manufacturer’s instruction. Briefly, cells were counted and suspended at a cellular density of 1x10^7^ cells/ml and incubated with an equal amount of 2X CytoTell Blue at 37°C for 30 minutes in the dark. Cells were then washed twice with media (RPMI 10% FBS) to remove excess dye. THP-1 cells were seeded in 12 well plates and treated with 200nM PMA for 8 h. THP-1 cells or primary monocytes were cultivated with effector cells previously lethally irradiated (20 min for a dosage of 12 grays, Precision X-Ray, 320KV X-rays). A well without effector cells was included for compensation and as a gating control.

Effectors cells (729.6 uninfected, 729.6 HTLV-1_WT_ or 729.6 HTLV-1_p12KO_) were stained with CFSE (Cayman Chemical) according to the manufacturer’s instructions and cells were washed twice with 20 ml of media (RPMI 10% FBS). Effector cells were added immediately to the well with bait cells (ratio 1:1) and left for 18 or 72 h. 10^6^ cells were kept in a separate well without THP-1 to be used for compensation and as a gating control. Cells were then collected and samples analyzed by flow cytometry (BD LSR II). Data were analyzed using FlowJo Version 10.6.

### Annexin V staining

Effector cells (729.6 HTLV-1_WT_ or 729.6 HTLV-1_p12KO_) were lethally irradiated (20 min for a dosage of 12 grays, Precision X-Ray, 320KV X-rays). Before 24, 48, and 72 h post-irradiation cells were stained with Annexin V, Alexa Fluor 647 conjugate (ThermoFisher Scientific). Briefly, cells were washed with PBS and 0.5x10^6^ cells were suspended in 1X Annexin V binding buffer (ThermoFisher Scientific). Alexa Fluor 647 conjugate (5 drops) was added to the cells and incubated at room temperature for 15 min in the dark. Cells were then washed with 2 ml of 1X Annexin V binding buffer. Cells were then collected and samples analyzed by flow cytometry (BD LSR II). Data were analyzed using FlowJo Version 10.6.

### CD47 staining

Effector cells (729.6 uninfected, 729.6 HTLV-1_WT_, and 729.6 HTLV-1_p12KO_) were washed with PBS. 10^6^ cells were stained with 10 μl of PE Mouse anti-human CD47 (BD Pharmingen, Franklin Lakes, NJ) together with a viability dye (Aqua fluorescent reactive dye, Invitrogen) for 30 min at room temperature in the dark. Cells were then collected and samples analyzed by flow cytometry (BD LSR II). Data were analyzed using FlowJo Version 10.6.

### Statistical analysis

The Wilcoxon test was used to compare the plasmatic cytokines in HTLV-1_p12KO_ and HTLV-1_WT_ infected animals to their own baseline and the Mann–Whitney two-tailed test was used to compare HTLV-1_p12KO_ and HTLV-1_WT_ infected animals. For *in vitro* experiments, the unpaired t-test was used for statistical evaluation.

## Supporting information

S1 Fig Study design of control groups: uninfected, IgG, and Liposome. (**A**) Schematic of study design: The animals in the α-CD8/NK control uninfected group were injected intravenously with M-T807R1 at 5 mg/kg per day for three days prior to inoculation with lethally irradiated uninfected 729.6 cells. The animals in the IgG WT group were injected intravenously with IgG control at 5mg/kg per day for three days prior to inoculation with lethally irradiated 729.6 HTLV-1_WT_ cells. Macaques in the Liposome WT group were injected with Liposome control at 20 mg/kg per day prior to inoculation with lethally irradiated 729.6 HTLV-1_WT_ cells. Black arrows represent the day of treatment (M-T807R1, IgG or the Liposome) and blue and gray arrows indicate the day of inoculation with the lethally irradiated 729.6 lymphoblastoid B-cell lines producing HTLV-1_WT_ or the parental uninfected 729.6 B cell lines, respectively. Absolute CD8^+^ T-cell numbers before (baseline), during (day 0), and after (weeks 2, 4, 6, 8, 10, 12, 16, 18, and 20) the administration of (**B**) M-T807R1 (**E**) or IgG in the groups inoculated with HTLV-1_WT_ or the parental uninfected 729.6 cell lines, respectively. (**C**) Frequency of NKG2A^+^ cells identified as Singlets/Live/CD45^+^/CD3^-^CD20^-^/NKG2A^+^ measured before (baseline), during (day 0), and after (every 2 weeks) CD8^+^ cell depletion in peripheral blood of the α-CD8/NK control uninfected group. (**D**) Frequency of NKG2A^+^CD16^+^ cells identified as Singlets/Live/CD45^+^/CD3^-^/CD20^-^/NKG2A^+^/CD16^+^ graphed at different timepoints in the PBMC of the α-CD8/NK control uninfected group. (**F**) Absolute monocyte cell numbers in the Liposome WT group before (baseline), during (day 0), and after (weeks 1, 3, 9, 15, and 21) the administration of Liposome and inoculation with HTLV-1_WT_. (**G**) Sera from the inoculated macaques belonging to α-CD8/NK control uninfected, IgG WT, and Liposome WT groups were assayed for reactivity to HTLV-1 antigens (weeks 2, 4, 8, 10, 12, and 21). The animal ID, inoculated viruses, and treatments are indicated above each sample. The week of sera collection is indicated below each Western blot strip. Positive amplification of either *gag* or *orf-I* in the blood throughout the course of the study is symbolized by (+); absence of amplification is symbolized by (-). (**H**) HTLV-1 p24 antibody titer in macaques belonging to α-CD8/NK WT, α-CD8 WT, and Clodronate WT groups at weeks 0, 12, and 21. Dilutions of 1:10, 1:100, 1:1000, and 1:3200 were used as reported.
(TIF)

S2 Fig Immunophenotype of control groups. Monocyte density. Density plot of CD45^+^CD3^+^CD4^+^, CD45^+^CD3^+^CD8^+^, CD45^+^CD14^+^ monocytes, CD45^+^CD16^+^ monocytes, CD45^+^CD20^+^, CD45^+^NKG2A^+^, and CD45^+^NKG2A^+^CD16^+^ cells at baseline, day 0, and weeks 2 and 21 of the animals included in the (**A**) α-CD8/NK control uninfected, (**B**) IgG HTLV-1_WT_, and (**C**) liposome HTLV-1_WT_ groups.
(TIF)

S3 Fig Immunophenotype of HTLV-1_WT_ inoculated animals. Monocyte density. Density plot of CD45^+^CD3^+^CD4^+^, CD45^+^CD3^+^CD8^+^, CD45^+^CD14^+^ monocytes, CD45^+^CD16^+^ monocytes, CD45^+^CD20^+^, CD45^+^NKG2A^+^, and CD45^+^NKG2A^+^CD16^+^ cells at baseline, day 0, and weeks 2 and 21 of the animals included in the (**A**) α-CD8/NK WT, (**B**) α-CD8 WT, and (**C**) Clodronate WT groups.
(TIF)

S4 Fig Immunophenotype of HTLV-1_p12KO_ inoculated animals. Monocyte density. Density plot of CD45^+^CD3^+^CD4^+^, CD45^+^CD3^+^CD8^+^, CD45^+^CD14^+^ monocytes, CD45^+^CD16^+^ monocytes, CD45^+^CD20^+^, CD45^+^NKG2A^+^, and CD45^+^NKG2A^+^CD16^+^ cells at baseline, day 0, and weeks 2 and 21 of the animals included in the (**A**) α-CD8/NK p12KO (**B**), α-CD8 p12KO, and (**C**) Clodronate p12KO groups.
(TIF)

S5 Fig Viral dissemination of HTLV-1_WT_ and HTLV-1_p12KO_. Summary of nested-PCR results using primers designed to amplify the (**A,B,C**) *gag* and the (**D,E,F**) *orf-I* genes performed on the genomic DNA isolated from the tissues listed below. Each slice of the pie chart represents an animal, and each segment represents a tissue collected at the time of the necropsy. From perimeter to center, segments represent: (1) PBMC, (2) Mesenteric LN, (3) Inguinal LN, (4) Axillary LN, (5) Lung LN, (6) Bone Marrow, (7) Spleen, (8) Thymus, (9) Lung, (10) Cortex, (11) Pons, (12) Jejunum, (13) Colon, (14) Ileum, and (15) Skin. Animals were divided based on the treatment and inoculated virus in 6 radial plots. (**A,D**) Control animals including α-CD8/NK control uninfected, IgG WT, and liposome WT groups. (**B,E**) HTLV-1_WT_, including α-CD8/NK WT, α-CD8 WT, and Clodronate WT groups. (**C,F**) HTLV-1_p12KO_ including α-CD8/NK p12KO, α-CD8 p12KO and Clodronate p12KO groups.
(TIF)

S6 Fig Co-cultivation of primary monocytes with WT or p12KO infected CD4^+^. (**A**) CD4 cells were isolated from two independent donors (ND1 and ND3) to establish CD4 virus-producing cells. This was done by co-culturing the isolated CD4 cells with irradiated 729.6 WT or p12KO producing cell lines. Once established, the CD4 cultures were adjusted for p19Gag supernatant production for co-cultivation with primary monocyte cultures. (**B**) Quantitative real-time PCR assay using the TaqMan probe was used to detect the provirus, as described previously (see Materials and Methods). The HTLV-1 copy number was measured with primers for the pX region. The RNase P gene was used as the endogenous reference in multiplex reactions. The viral levels are presented as the number of copies of HTLV-1 per 100 copies of the RNase P gene. (**C,D**) Monocytes were co-cultivated with primary infected CD4^+^ for 24 h; cells were then washed every day to remove the infected HTLV-1_WT_ and HTLV-1_p12KO_ CD4^+^ from the culture and maintained in culture for a total of four days in DMEM media supplemented with 10% FBS and 10% human AB sera. Production of p19Gag in the supernatant was measured at days 2, 3, and 4, as illustrated in the figure. (**E**) Four days following co-cultivation, monocytes were stained to demonstrate the purity of the culture by flow cytometry. (**F**) Cells were stained with CD3, CD14, CD16 antibodies, and viability dye. Percentage of CD3^-^CD14^+^ and classical (CD14^+^CD16^-^), intermediate (CD14^+^CD16^+^), and non-classical (CD14^-^CD16^+^) monocyte subsets were analyzed. Remaining free CD3^+^ cells were negligible in most cases. Graphs represent results from 4 independent experiments. (**G**) Cytokines and chemokines measured in the cryopreserved supernatants from monocytes isolated from four different donors at three days post-co-cultivation with uninfected HTLV-1_WT_ and HTLV-1_p12KO_ CD4^+^.
(TIF)

S7 Fig Viral integration in MHC-I-matched and unmatched monocytes and CD4^+^. (**A**) Confocal microscopy of monocytes two days post co-cultivation with infected CD4^+^. Prior to co-cultivation, CD4^+^ infected cells were labeled with Cell Tracker Blue (blue) and the plasma membrane of monocytes labeled with WGA594 (red). After 24 h, cells were washed three times with PBS (scale bar = 10mm), mixed, and cultured an additional 24 h before fixation (scale bar = 10mm). (**B,C**) Genomic DNA was extracted from monocytes 3 days post co-cultivation with infected CD4^+^. High-throughput sequencing (HTS) was used to map viral integration sites. Pie charts illustrate the relative abundance of HTLV-1_WT_ and HTLV-1_p12KO_ provirus in monocytes following co-cultivation with matched and unmatched infected CD4^+^. Each slice represents a unique integration site, with size corresponding to relative abundance. The analysis shows monocytes following co-cultivation with infected CD4^+^ isolated from (**B**) MHC-I-matched CD4^+^/monocytes, both from ND1, or (**C**) MHC-I unmatched CD4^+^ from ND1 and monocytes from ND3.
(TIF)

S8 Fig Efferocytosis of HTLV-1 infected cells. (**A**) Efferocytosis assay using rhesus macaque monocytes co-cultivated with WT infected cells. The bait cells, primary monocytes isolated by adherence, were labeled with Far Red co-cultivated for 18 h with effector cells (729.6 HTLV-1_WT_) previously stained with CFSE and lethally γ-irradiated. A well without effector cells was included for compensation and as a gating control. (**B**) Percentage of engulfment cells (Far Red and CFSE positive cells) were graphed. (**C**) Efferocytosis assay of THP-1 cells co-cultivated with WT and p12KO infected cells. The bait cells, THP-1, were labeled with CytoTell Blue. Cells were then seeded in 12 well plates and treated with PMA. THP-1 cells were cultivated for 72 h with effector cells (729.6 HTLV-1_WT_ or 729.6 HTLV-1_p12KO_ cells) previously stained with CFSE and lethally γ-irradiated. A well without effector cells was included for compensation and as a gating control. (**D**) 729.6 HTLV-1_WT_ and 729.6 HTLV-1_p12KO_ cells were lethally γ-irradiated. Apoptosis was assessed by Annexin V staining of samples before irradiation (0 h) and 24, 48, or 72 h post irradiation. The percentage of apoptotic cells was graphed for WT (blue) and p12KO (red) cells from three independent experiments. No significant difference was noted.
(TIF)

S1 Table Animal prior history. Profiles of animals and their individual treatment histories.
(XLSX)

S2 Table Viral DNA status in immune tissues of all animals at the time of euthanasia. Viral DNA status of all animals measured in PBMCs, bone marrow (BM), and mesenteric (MS), inguinal (Ing), axillary (Ax), and lung lymph nodes. Positive amplification by nested PCR is symbolized by (+), and the absence of amplification by (-). Animals ZJ22, ZI51, HPM, and ZJ58 were not sacrificed at the time of this study so samples were not available.
(XLSX)

S3 Table Antibodies used for non-human primate flow cytometry. Antibodies used for non-human primate flow cytometry, including clones, fluorochromes, and source.
(XLSX)
