# Supplementary figures and images for: NK cells and monocytes modulate primary HTLV-1 infection
Source: PLoS Pathog. 2022 Apr 4;18(4):e1010416. doi: 10.1371/journal.ppat.1010416 (PMC9022856; doi:10.1371/journal.ppat.1010416)

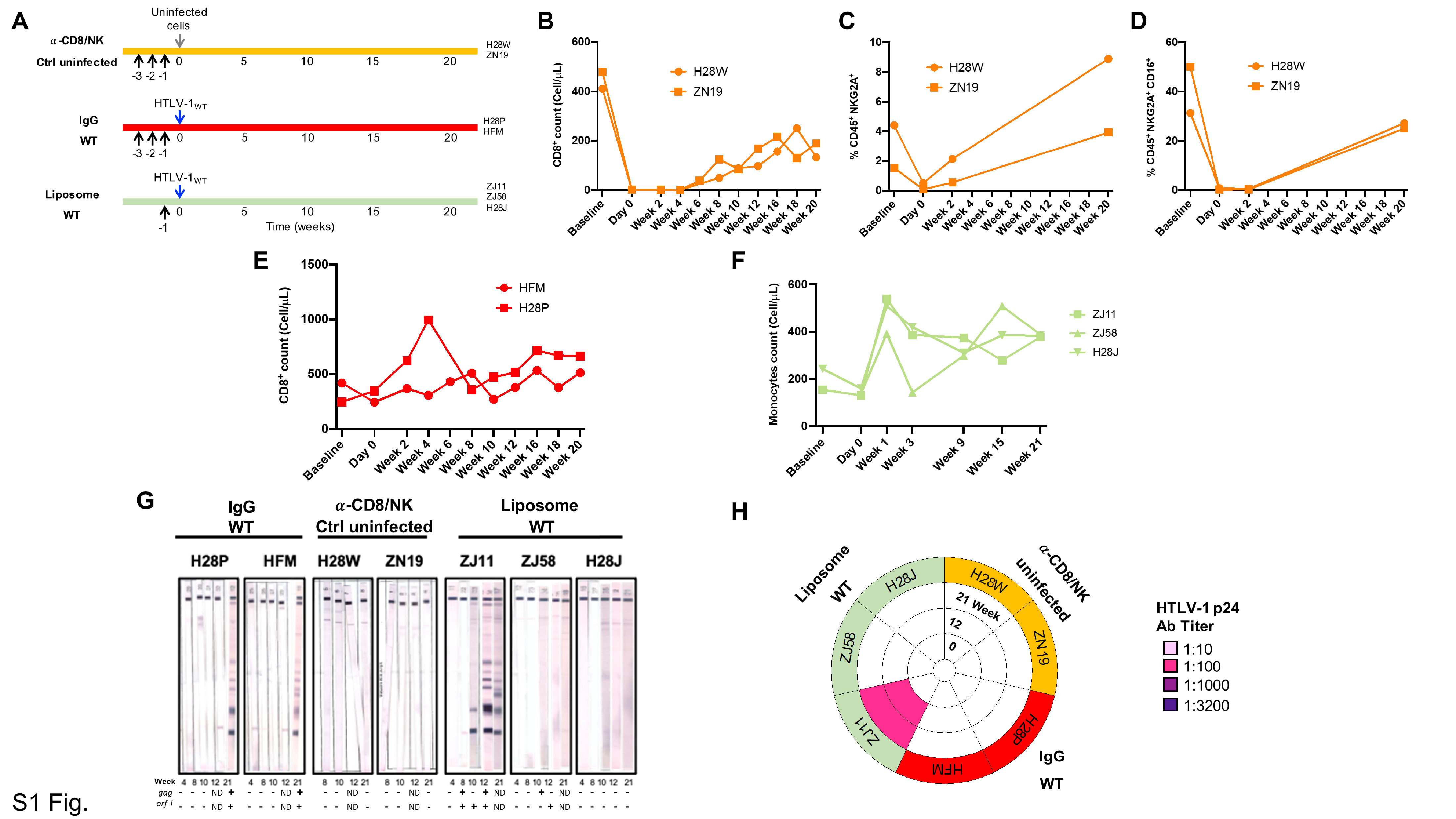

Supplement: S1 Fig — (A) Schematic of study design: The animals in the α-CD8/NK control uninfected group were injected intravenously with M-T807R1 at 5 mg/kg per day for three days prior to inoculation with lethally irradiated uninfected 729.6 cells. The animals in the IgG WT group were injected intravenously with IgG control at 5mg/kg per day for three days prior to inoculation with lethally irradiated 729.6 HTLV-1WT cells. Macaques in the Liposome WT group were injected with Liposome control at 20 mg/kg per day prior to inoculation with lethally irradiated 729.6 HTLV-1WT cells. Black arrows represent the day of treatment (M-T807R1, IgG or the Liposome) and blue and gray arrows indicate the day of inoculation with the lethally irradiated 729.6 lymphoblastoid B-cell lines producing HTLV-1WT or the parental uninfected 729.6 B cell lines, respectively. Absolute CD8+ T-cell numbers before (baseline), during (day 0), and after (weeks 2, 4, 6, 8, 10, 12, 16, 18, and 20) the administration of (B) M-T807R1 (E) or IgG in the groups inoculated with HTLV-1WT or the parental uninfected 729.6 cell lines, respectively. (C) Frequency of NKG2A+ cells identified as Singlets/Live/CD45+/CD3-CD20-/NKG2A+ measured before (baseline), during (day 0), and after (every 2 weeks) CD8+ cell depletion in peripheral blood of the α-CD8/NK control uninfected group. (D) Frequency of NKG2A+CD16+ cells identified as Singlets/Live/CD45+/CD3-/CD20-/NKG2A+/CD16+ graphed at different timepoints in the PBMC of the α-CD8/NK control uninfected group. (F) Absolute monocyte cell numbers in the Liposome WT group before (baseline), during (day 0), and after (weeks 1, 3, 9, 15, and 21) the administration of Liposome and inoculation with HTLV-1WT. (G) Sera from the inoculated macaques belonging to α-CD8/NK control uninfected, IgG WT, and Liposome WT groups were assayed for reactivity to HTLV-1 antigens (weeks 2, 4, 8, 10, 12, and 21). The animal ID, inoculated viruses, and treatments are indicated above each sample. The week of sera [file ppat.1010416.s001.TIF]

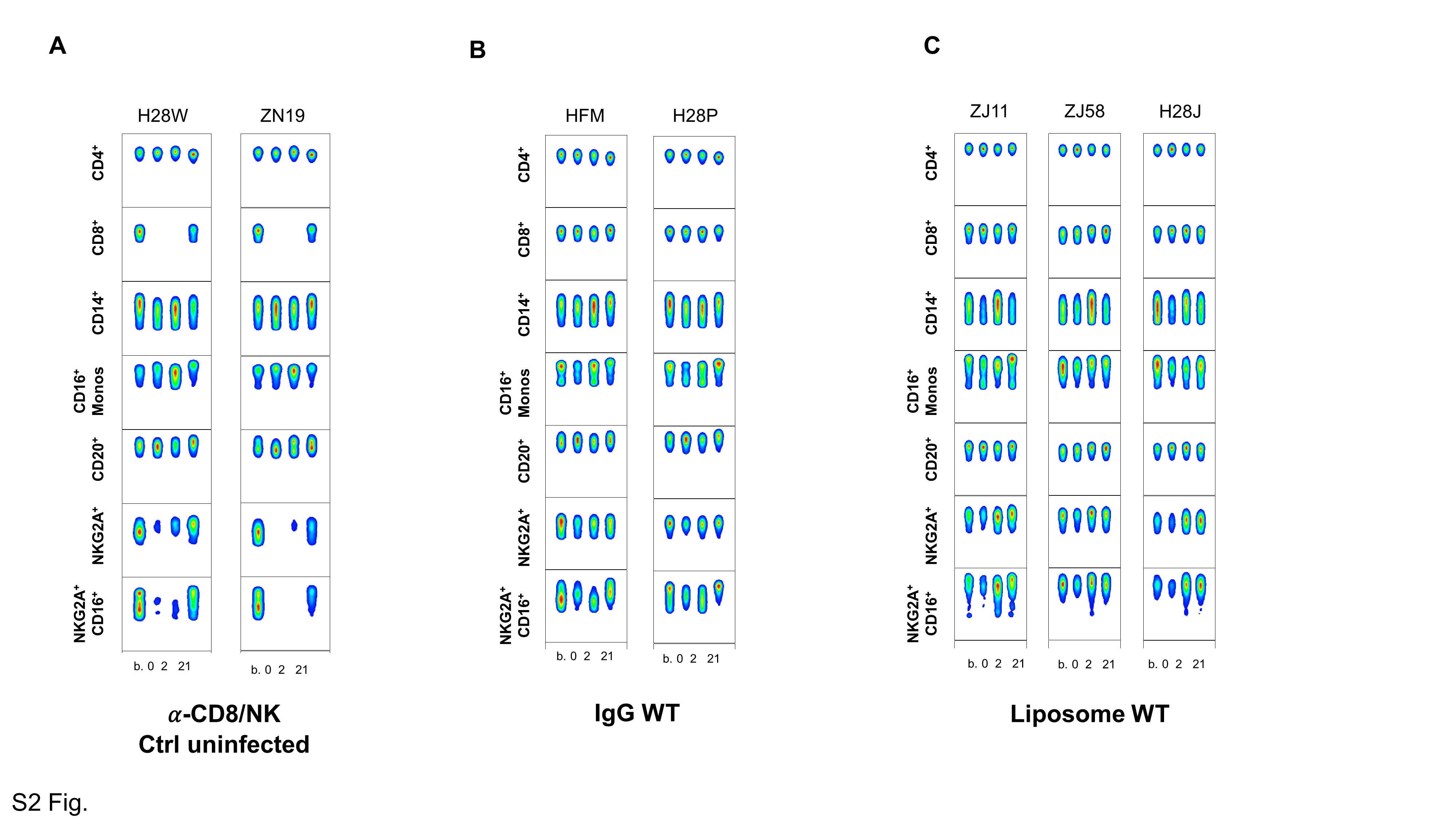

Supplement: S2 Fig — Monocyte density. Density plot of CD45+CD3+CD4+, CD45+CD3+CD8+, CD45+CD14+ monocytes, CD45+CD16+ monocytes, CD45+CD20+, CD45+NKG2A+, and CD45+NKG2A+CD16+ cells at baseline, day 0, and weeks 2 and 21 of the animals included in the (A) α-CD8/NK control uninfected, (B) IgG HTLV-1WT, and (C) liposome HTLV-1WT groups. (TIF) [file ppat.1010416.s002.TIF]

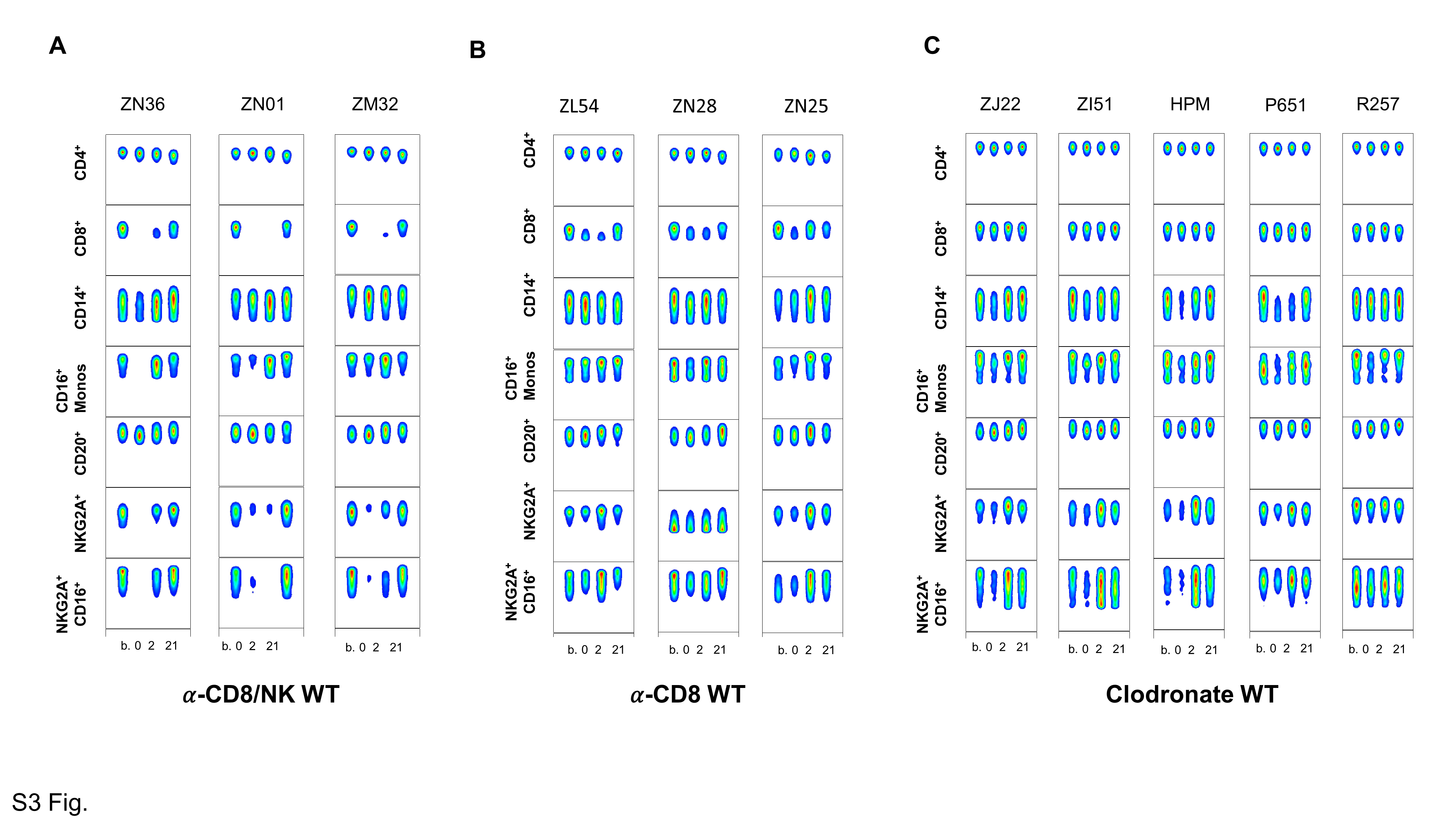

Supplement: S3 Fig — Monocyte density. Density plot of CD45+CD3+CD4+, CD45+CD3+CD8+, CD45+CD14+ monocytes, CD45+CD16+ monocytes, CD45+CD20+, CD45+NKG2A+, and CD45+NKG2A+CD16+ cells at baseline, day 0, and weeks 2 and 21 of the animals included in the (A) α-CD8/NK WT, (B) α-CD8 WT, and (C) Clodronate WT groups. (TIF) [file ppat.1010416.s003.TIF]

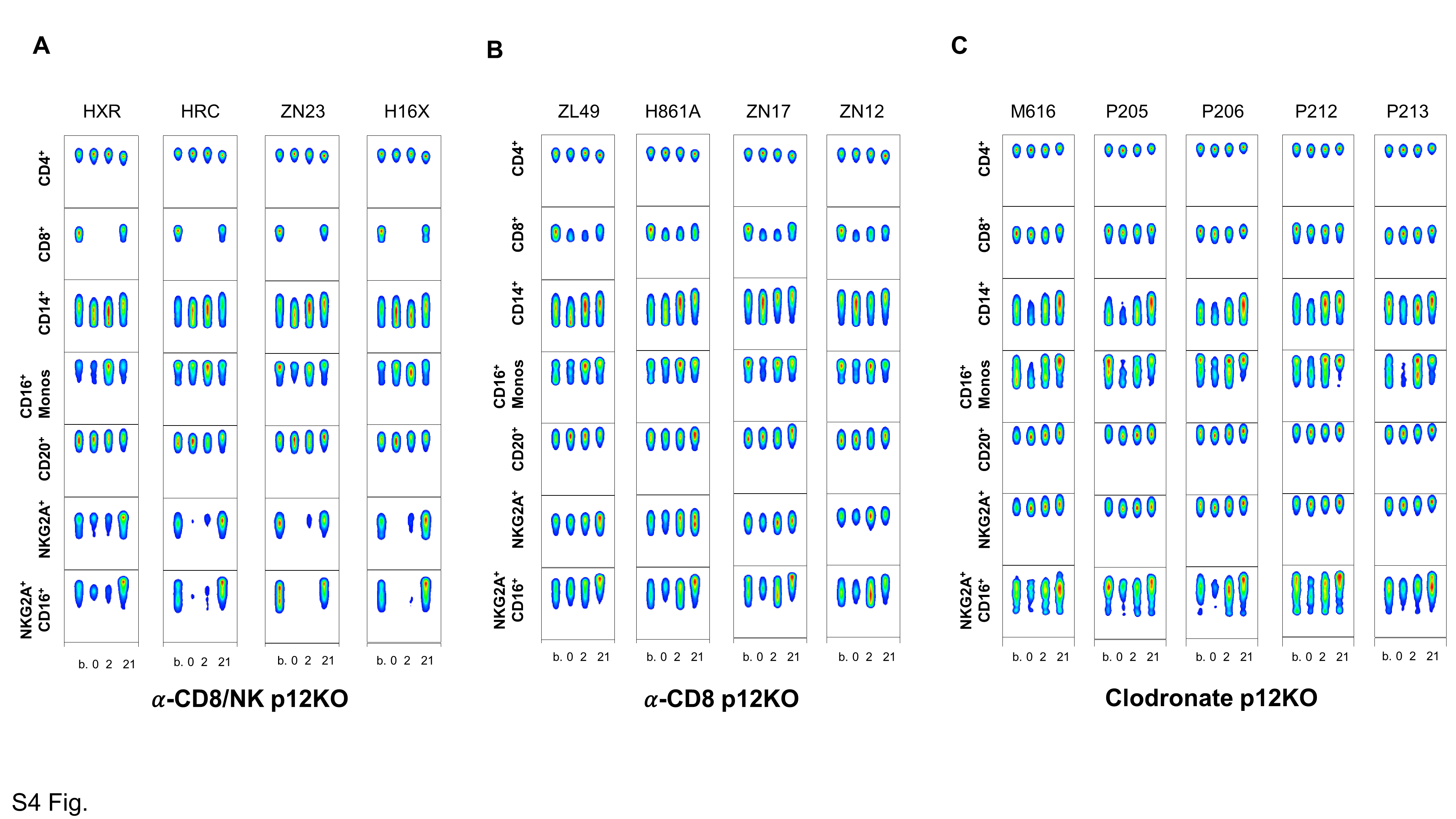

Supplement: S4 Fig — Monocyte density. Density plot of CD45+CD3+CD4+, CD45+CD3+CD8+, CD45+CD14+ monocytes, CD45+CD16+ monocytes, CD45+CD20+, CD45+NKG2A+, and CD45+NKG2A+CD16+ cells at baseline, day 0, and weeks 2 and 21 of the animals included in the (A) α-CD8/NK p12KO (B), α-CD8 p12KO, and (C) Clodronate p12KO groups. (TIF) [file ppat.1010416.s004.TIF]

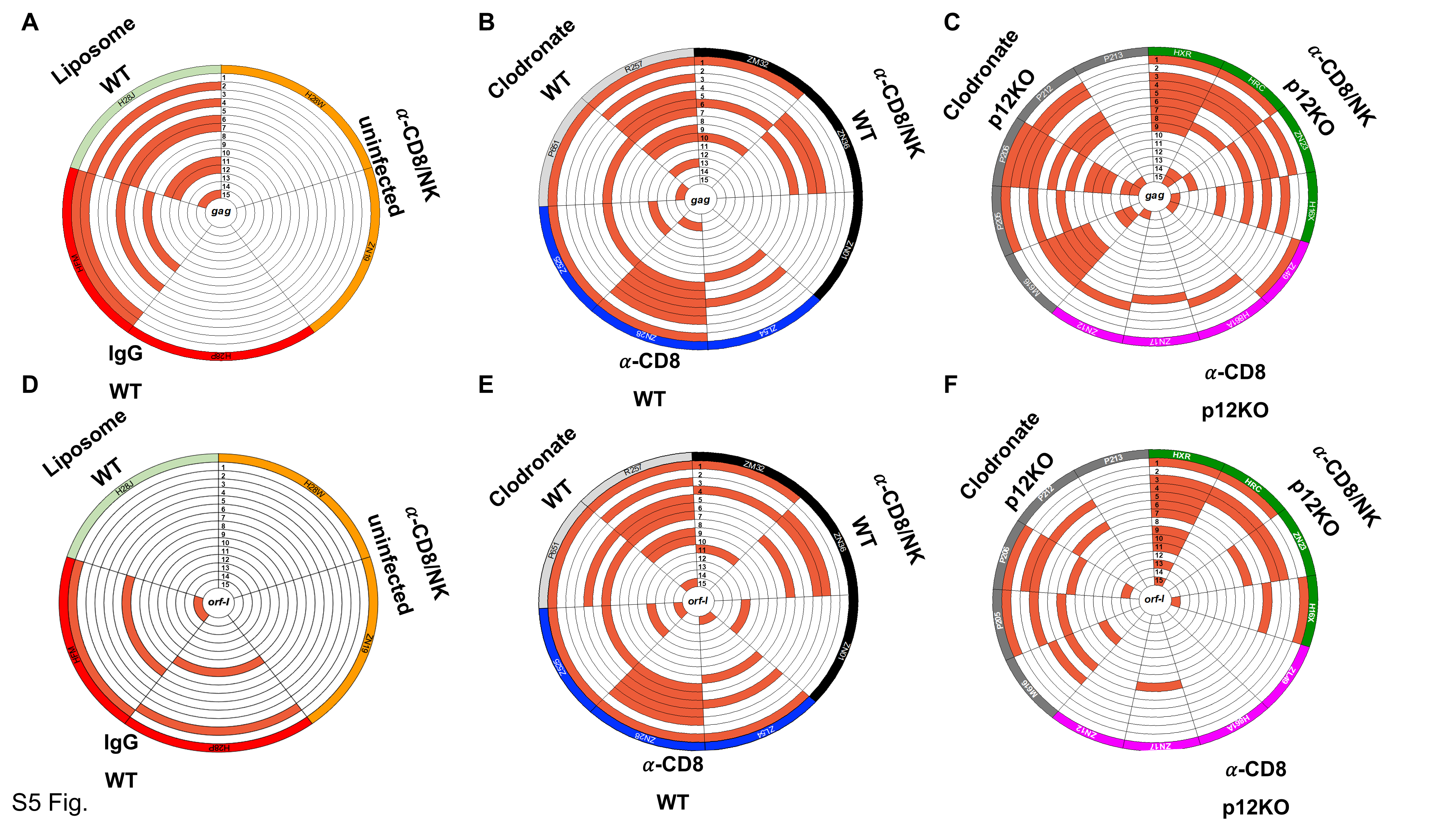

Supplement: S5 Fig — Summary of nested-PCR results using primers designed to amplify the (A,B,C) gag and the (D,E,F) orf-I genes performed on the genomic DNA isolated from the tissues listed below. Each slice of the pie chart represents an animal, and each segment represents a tissue collected at the time of the necropsy. From perimeter to center, segments represent: (1) PBMC, (2) Mesenteric LN, (3) Inguinal LN, (4) Axillary LN, (5) Lung LN, (6) Bone Marrow, (7) Spleen, (8) Thymus, (9) Lung, (10) Cortex, (11) Pons, (12) Jejunum, (13) Colon, (14) Ileum, and (15) Skin. Animals were divided based on the treatment and inoculated virus in 6 radial plots. (A,D) Control animals including α-CD8/NK control uninfected, IgG WT, and liposome WT groups. (B,E) HTLV-1WT, including α-CD8/NK WT, α-CD8 WT, and Clodronate WT groups. (C,F) HTLV-1p12KO including α-CD8/NK p12KO, α-CD8 p12KO and Clodronate p12KO groups. (TIF) [file ppat.1010416.s005.TIF]

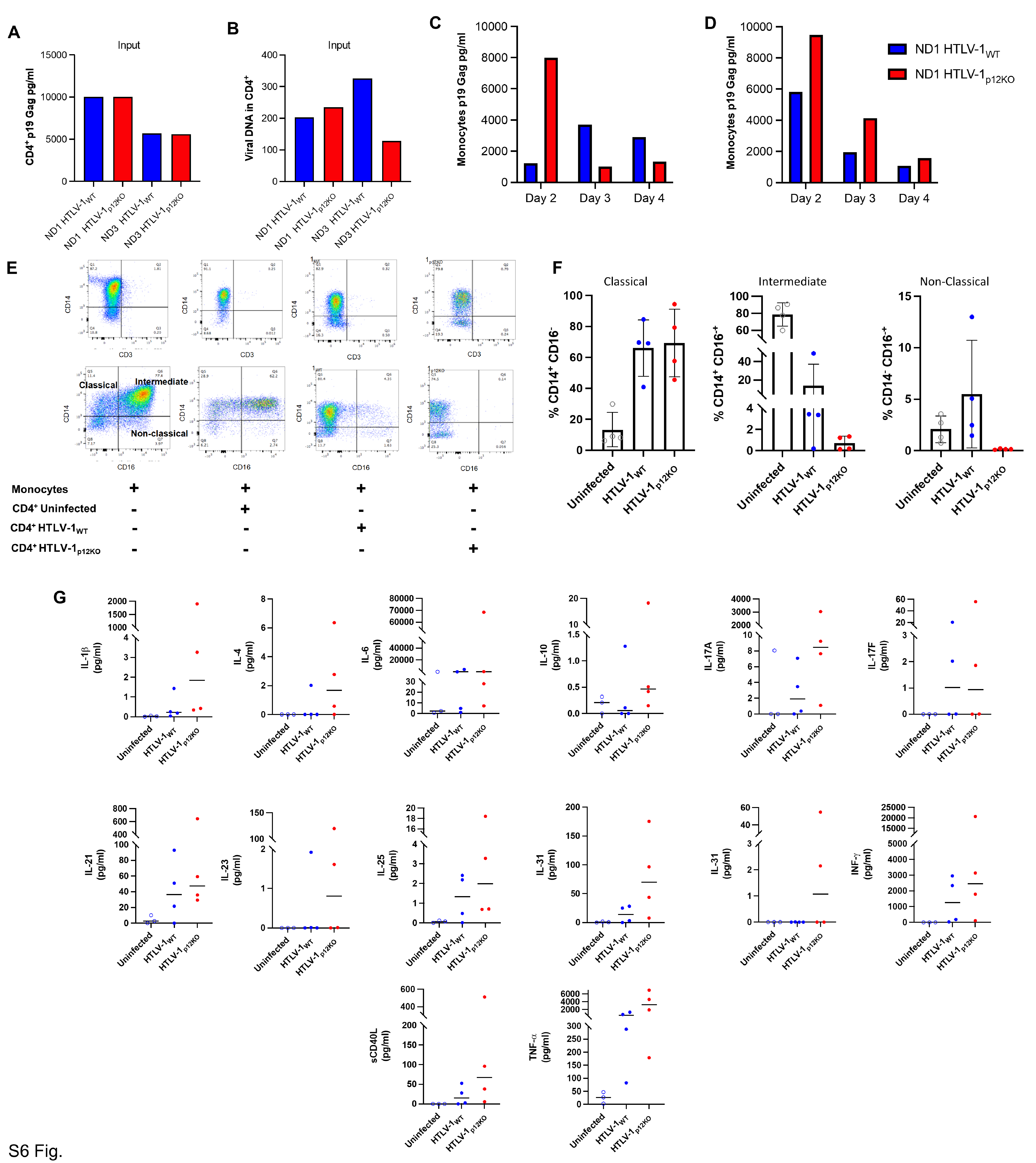

Supplement: S6 Fig — (A) CD4 cells were isolated from two independent donors (ND1 and ND3) to establish CD4 virus-producing cells. This was done by co-culturing the isolated CD4 cells with irradiated 729.6 WT or p12KO producing cell lines. Once established, the CD4 cultures were adjusted for p19Gag supernatant production for co-cultivation with primary monocyte cultures. (B) Quantitative real-time PCR assay using the TaqMan probe was used to detect the provirus, as described previously (see Materials and Methods). The HTLV-1 copy number was measured with primers for the pX region. The RNase P gene was used as the endogenous reference in multiplex reactions. The viral levels are presented as the number of copies of HTLV-1 per 100 copies of the RNase P gene. (C,D) Monocytes were co-cultivated with primary infected CD4+ for 24 h; cells were then washed every day to remove the infected HTLV-1WT and HTLV-1p12KO CD4+ from the culture and maintained in culture for a total of four days in DMEM media supplemented with 10% FBS and 10% human AB sera. Production of p19Gag in the supernatant was measured at days 2, 3, and 4, as illustrated in the figure. (E) Four days following co-cultivation, monocytes were stained to demonstrate the purity of the culture by flow cytometry. (F) Cells were stained with CD3, CD14, CD16 antibodies, and viability dye. Percentage of CD3-CD14+ and classical (CD14+CD16-), intermediate (CD14+CD16+), and non-classical (CD14-CD16+) monocyte subsets were analyzed. Remaining free CD3+ cells were negligible in most cases. Graphs represent results from 4 independent experiments. (G) Cytokines and chemokines measured in the cryopreserved supernatants from monocytes isolated from four different donors at three days post-co-cultivation with uninfected HTLV-1WT and HTLV-1p12KO CD4+. (TIF) [file ppat.1010416.s006.tif]

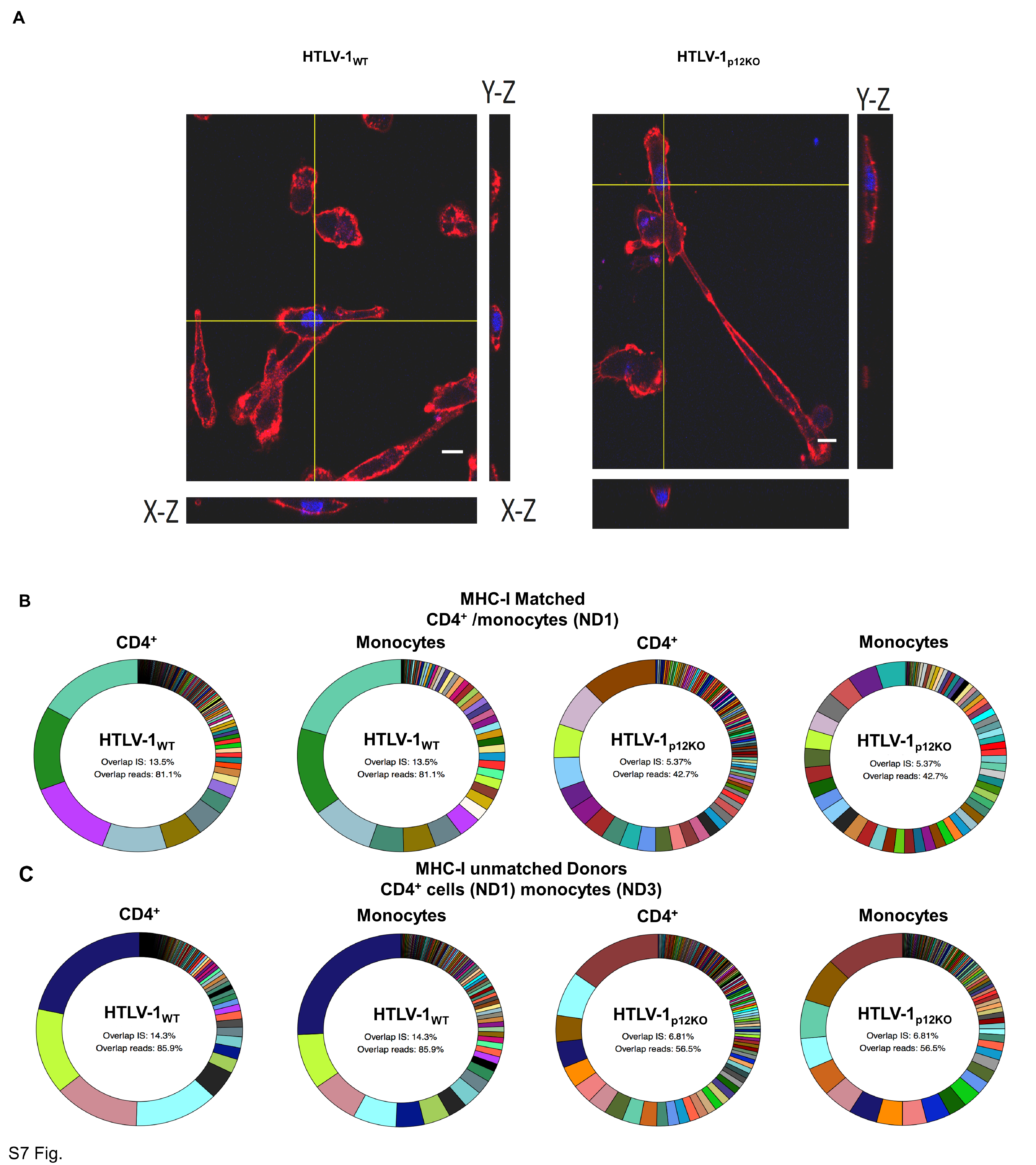

Supplement: S7 Fig — (A) Confocal microscopy of monocytes two days post co-cultivation with infected CD4+. Prior to co-cultivation, CD4+ infected cells were labeled with Cell Tracker Blue (blue) and the plasma membrane of monocytes labeled with WGA594 (red). After 24 h, cells were washed three times with PBS (scale bar = 10mm), mixed, and cultured an additional 24 h before fixation (scale bar = 10mm). (B,C) Genomic DNA was extracted from monocytes 3 days post co-cultivation with infected CD4+. High-throughput sequencing (HTS) was used to map viral integration sites. Pie charts illustrate the relative abundance of HTLV-1WT and HTLV-1p12KO provirus in monocytes following co-cultivation with matched and unmatched infected CD4+. Each slice represents a unique integration site, with size corresponding to relative abundance. The analysis shows monocytes following co-cultivation with infected CD4+ isolated from (B) MHC-I-matched CD4+/monocytes, both from ND1, or (C) MHC-I unmatched CD4+ from ND1 and monocytes from ND3. (TIF) [file ppat.1010416.s007.tif]

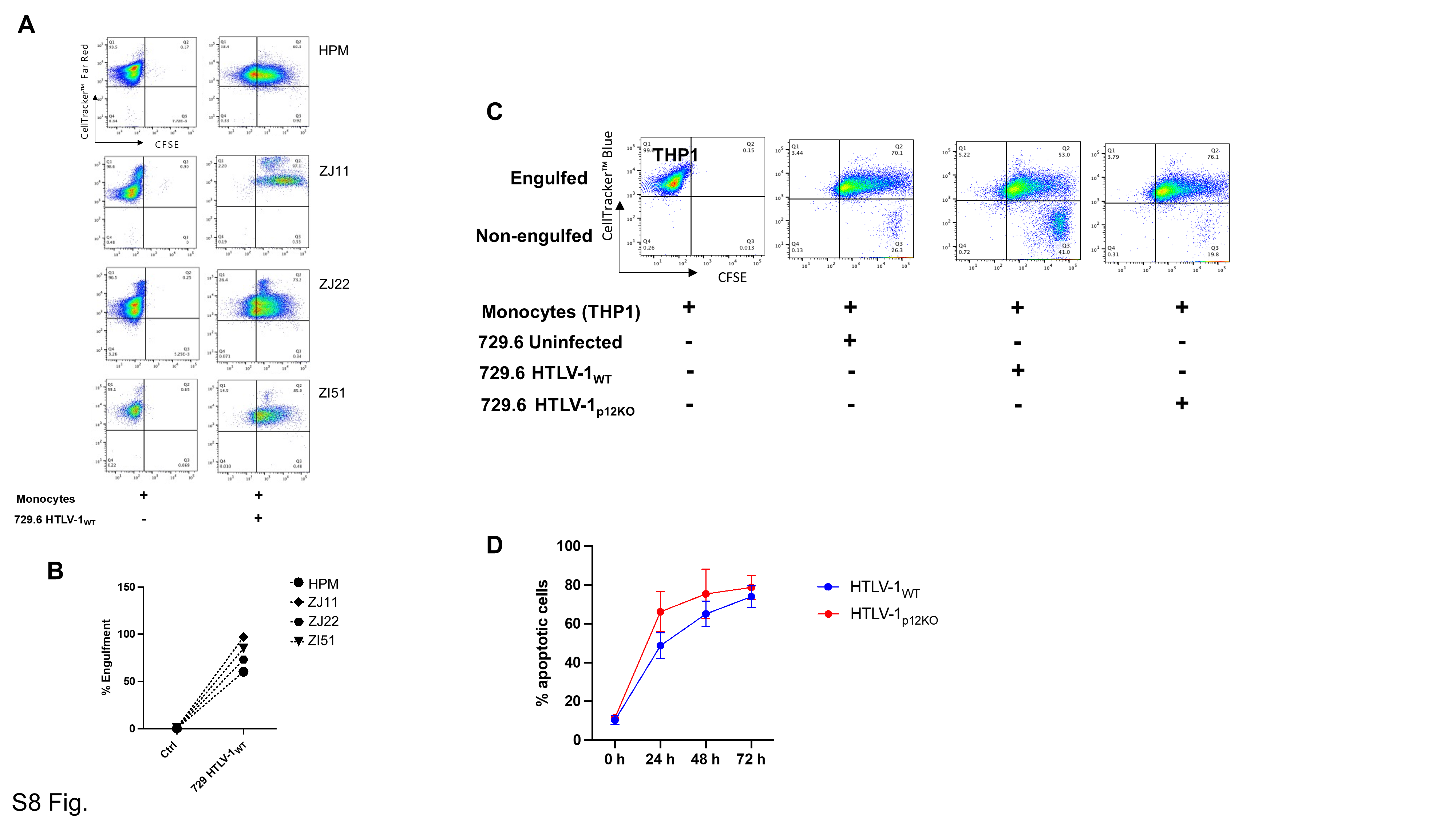

Supplement: S8 Fig — (A) Efferocytosis assay using rhesus macaque monocytes co-cultivated with WT infected cells. The bait cells, primary monocytes isolated by adherence, were labeled with Far Red co-cultivated for 18 h with effector cells (729.6 HTLV-1WT) previously stained with CFSE and lethally γ-irradiated. A well without effector cells was included for compensation and as a gating control. (B) Percentage of engulfment cells (Far Red and CFSE positive cells) were graphed. (C) Efferocytosis assay of THP-1 cells co-cultivated with WT and p12KO infected cells. The bait cells, THP-1, were labeled with CytoTell Blue. Cells were then seeded in 12 well plates and treated with PMA. THP-1 cells were cultivated for 72 h with effector cells (729.6 HTLV-1WT or 729.6 HTLV-1p12KO cells) previously stained with CFSE and lethally γ-irradiated. A well without effector cells was included for compensation and as a gating control. (D) 729.6 HTLV-1WT and 729.6 HTLV-1p12KO cells were lethally γ-irradiated. Apoptosis was assessed by Annexin V staining of samples before irradiation (0 h) and 24, 48, or 72 h post irradiation. The percentage of apoptotic cells was graphed for WT (blue) and p12KO (red) cells from three independent experiments. No significant difference was noted. (TIF) [file ppat.1010416.s008.TIF]
